# Mapping of Sensory Nerve Subsets within the Vagal Ganglia and the Brainstem Using Reporter Mice for Pirt, TRPV1, 5-HT3, and Tac1 Expression

**DOI:** 10.1523/ENEURO.0494-19.2020

**Published:** 2020-03-31

**Authors:** Seol-Hee Kim, Stephen H. Hadley, Mikayla Maddison, Mayur Patil, Byeong Cha, Marian Kollarik, Thomas E. Taylor-Clark

**Affiliations:** Molecular Pharmacology & Physiology, Morsani College of Medicine, University of South Florida, Tampa, Florida 33612

**Keywords:** mapping, medulla, nociception, nucleus tractus solitarius, vagal afferents

## Abstract

Vagal afferent sensory nerves, originating in jugular and nodose ganglia, are composed of functionally distinct subsets whose activation evokes distinct thoracic and abdominal reflex responses. We used Cre-expressing mouse strains to identify specific vagal afferent populations and map their central projections within the brainstem. We show that Pirt is expressed in virtually all vagal afferents; whereas, 5-HT3 is expressed only in nodose neurons, with little expression in jugular neurons. Transient receptor potential vanilloid 1 (TRPV1), the capsaicin receptor, is expressed in a subset of small nodose and jugular neurons. Tac1, the gene for tachykinins, is expressed predominantly in jugular neurons, some of which also express TRPV1. Vagal fibers project centrally to the nucleus tractus solitarius (nTS), paratrigeminal complex, area postrema, and to a limited extent the dorsal motor nucleus of the vagus. nTS subnuclei preferentially receive projections by specific afferent subsets, with TRPV1^+^ fibers terminating in medial and dorsal regions predominantly caudal of obex, whereas TRPV1^−^ fibers terminate in ventral and lateral regions throughout the rostral–caudal aspect of the medulla. Many vagal Tac1^+^ afferents (mostly derived from the jugular ganglion) terminate in the nTS. The paratrigeminal complex was the target of multiple vagal afferent subsets. Importantly, lung-specific TRPV1^+^ and Tac1^+^ afferent terminations were restricted to the caudal medial nTS, with no innervation of other medulla regions. In summary, this study identifies the specific medulla regions innervated by vagal afferent subsets. The distinct terminations provide a neuroanatomic substrate for the diverse range of reflexes initiated by vagal afferent activation.

## Significance Statement

Vagal afferents transmit sensory information from visceral organs to the brainstem, where their activity alters sensation and visceral reflexes. Vagal afferents are composed of distinct subsets, which serve distinct functions. Little is known of the neuroanatomy of central projections of distinct vagal subsets; thus, there remains an incomplete understanding of how visceral events evoke appropriate behavioral and reflex responses. This precludes rationally developed pharmacological or electroceutical interventions to modify aberrant sensations/reflexes. Here, we used cell-specific reporter expression to identify the brainstem pathways of distinct vagal afferent subsets. We show that TRPV1^+^ vagal afferents innervate ipsilateral and contralateral dorsal/medial nTS subnuclei and the ipsilateral paratrigeminal complex, whereas TRPV1^−^ vagal afferents innervate the ipsilateral rostral/ventral/lateral nTS subnuclei and the ipsilateral paratrigeminal complex.

## Introduction

Afferent sensory nerves transmit information regarding physical, mechanical, thermal, and chemical conditions within peripheral tissues to central networks within the brainstem and spinal cord. Thus, afferent signaling regulates homeostatic physiological mechanisms, initiates protective reflexes, causes sensation, and contributes to emotional and behavioral responses. Most sensory nerves are projected from soma that reside in spinal dorsal root ganglia (DRGs) or from ganglia associated with cranial nerve V (trigeminal nerve and ganglia), VII (facial nerve and geniculate ganglia), VIII (vestibular nerve and ganglia), IX (glossopharyngeal nerve and petrosal ganglia), and X (vagal nerve and ganglia, composed of the distinct nodose and jugular ganglia). Sensory nerves are heterogeneous with respect to their sensitivity to specific stimuli and the functional outcome of their activation, largely due to differences in anatomic location, neuronal structure, and protein expression (Thoren, 1979; Ricco et al., 1996; Zhuo et al., 1997; Mazzone and Canning, 2002; Carr and Undem, 2003; Mazzone et al., 2005; Yu et al., 2005; Nassenstein et al., 2010; Chang et al., 2015; Usoskin et al., 2015; Wang et al., 2017; Chou et al., 2018; Kupari et al., 2019). One defining characteristic of a major subset of sensory afferents is their ability to discriminate noxious stimuli (due to the selective expression of specific receptors) from non-noxious stimuli (Patapoutian et al., 2009). These sensory afferents, which are often small-diameter unmyelinated neurons, have been termed “nociceptors” (Sherrington, 1906). Activation of nociceptors evokes aversive responses, which can include defensive reflexes (e.g., limb withdrawal or cough) or sensations (e.g., pain or itch) depending on the afferent subset. Sensory nerves that are only able to discriminate non-noxious stimuli have been termed “non-nociceptors.”

In the vagal system, nodose neurons are embryologically derived from the placodes and jugular neurons are derived from the neural crest. Despite their close proximity in the adult (in mice, the ganglia are anatomically fused together), these distinct groups express distinct patterns of developmental pathways, neurotrophin receptors, neurotransmitters, and receptors for endogenous/exogenous stimuli (Nassenstein et al., 2010; Lieu et al., 2011; Wang et al., 2017; Kupari et al., 2019). Much is known of the functional roles that specific vagal afferents play in the homeostatic and defensive control of thoracic and abdominal organs (Thoren, 1979; Carr and Undem, 2003; Kubin et al., 2006; Robinson and Gebhart, 2008). Vagal afferents have been extensively studied using electrophysiology, and many of the specific transduction mechanisms activated by endogenous and exogenous stimuli have been elucidated. However, less is known of the neuroanatomy of specific vagal afferent subsets. Vagal ganglia have been probed using *in situ* hybridization and immunohistochemistry, but these techniques are either unsuitable for determining terminal anatomy/structure or lack selectivity and robust signal-to-noise ratios for determining terminal anatomy/structure. Studies using tracers such as horseradish peroxidase or the fluorescent compound DiI found that the majority of vagal afferents terminate within the large and diverse nucleus tractus solitarius (nTS) region of the medulla (Kalia and Mesulam, 1980; Kalia and Sullivan, 1982), but these are nonspecific tracers that do not identify specific afferent subsets. The aim of this current study was to improve our understanding of the central pathways of specific afferent subsets that regulate distinct reflexes.

Here, we have used the Cre/lox system (Yu and Bradley, 2001) to identify specific afferent subsets and to map their neuroanatomy. We have used four Cre-expressing strains (Pirt-Cre, TRPV1-Cre, 5-HT3-Cre, and Tac1-Cre) to identify specific vagal afferent populations and to map their central projections within the medulla. Pirt is a protein that regulates the function of transient receptor potential (TRP) channels and is expressed in the vast majority of peripheral sensory afferent neurons but not in other cell types (Patel et al., 2011). TRP vanilloid 1 (TRPV1) is an ion channel that is activated by capsaicin, heat, and extracellular acidification (Caterina et al., 1997) and is expressed in the majority of vagal C-fiber afferents (Yu et al., 2005; Nassenstein et al., 2010; Wang et al., 2017), previously described as nociceptors based on their ability to discriminate noxious stimuli (although there is little evidence to suggest that they evoke pain when activated). Based on sensitivity to 5-HT3 agonists, 5-HT3 is expressed in nodose neurons but not in jugular neurons (Chuaychoo et al., 2005; Potenzieri et al., 2012). Tac1 is the gene that encodes the precursor to the neuropeptide substance P (Carter and Krause, 1990). Substance P is expressed widely in jugular TRPV1^+^ neurons but has limited expression in nodose neurons (Ricco et al., 1996; Undem et al., 2004; Nassenstein et al., 2010).

## Materials and Methods

### Animals

All procedures were performed in accordance with the animal protocol approved by the Institutional Animal Care and Use Committee. The following four Cre strains were used: (1) the knock-in TRPV1-Cre [B6.129X1-Trpv1tm1(cre)Bbm/J; catalog #017769, The Jackson Laboratory]; (2) the knock-in Tac1-Cre [B6.129S-Tac1<tm1.1(cre)Hze/J; catalog #201877, The Jackson Laboratory]; (3) the knock-in Pirt-Cre [Pirttm3.1(cre)Xzd; gift from Dr Xinzhong Dong, Johns Hopkins University (Baltimore, MD); Kim et al., 2016]; and (4) the transgenic 5-HT3-Cre [B6.FVB(Cg)-Tg(Htr3a-cre)NO152Gsat/Mmucd; catalog #037089-UCD, Mutant Mouse Resource & Research Centers]. In most cases, the Cre strains were crossed with the ROSA26-loxP-STOP-loxP-tdTomato (tdT) mice [B6.Cg-Gt(ROSA)26Sortm9(CAG-tdTomato)Hze/J; catalog #007909, The Jackson Laboratory] to produce TRPV1-tdT, Tac1-tdT, Pirt-tdT, and 5-HT3-tdT mice with cell-specific expression of tdT via Cre recombination. Specific alleles were confirmed by genotyping per developer instructions. Both male and female mice (6–8 weeks old) were used for experiments. Offspring were weaned at 21 postnatal days, and up to four littermates were housed per cage under normal conditions (20°C; 12 h dark/light cycle). Mice were provided with standard rodent chow and water *ad libitum*.

### Unilateral intraganglionic injection of adeno-associated virus 9 for vagal afferent nerve tracing

The following adeno-associated virus 9 (AAV9) were purchased from Addgene: (1) AAV9-Flex-GFP, with cre-sensitive enhanced green fluorescent protein (GFP) expression under the control of a cytomegalovirus enhancer fused to the chicken β-actin (CAG) promoter and a woodchuck hepatitis virus post-transcriptional regulatory element (WPRE; 1.9 × 10^13^ GC/ml, #510502); (2) AAV9-GFP, with constitutively active GFP expression under the control of the human synapsin promoter and a WPRE [1.9 × 10^13^ genome copies (GC)/ml; #50465]; and (3) AAV9-Flex-tdT, with cre-sensitive tdTomato expression under the control of a CAG promoter and a WPRE (2.1 × 10^13^ GC/ml; #28306). Mice were anesthetized with a mixture of ketamine (50 mg/kg) and dexmedetomidine (0.5 mg/kg) via intraperitoneal injection. Approximately 2 cm of incision was made over a shaved superficial portion of the masseter muscle area. The skin was retracted from the exposed muscle area using clamps. After identifying the upper part of the trachea, the vagus nerve was located. Care was taken not to touch the vagus nerve directly with the forceps. The vagus nerve path leading into the ear bone was followed. The vagus nerve sits between the common carotid artery and the anterior laryngeal nerve. The separation between these three structures was created using a pointed cotton tip applicator. Care was taken not to apply too much pressure or rupture the artery. The vagus nerve ends into the distal part of the nodose ganglia (distinct from the superior cervical ganglion). The vagal nodose ganglia were then carefully exposed by scraping the muscle and membrane tissue using blunt-tipped instruments. Sterile cotton-tipped applicators and cotton pads were used to control bleeding throughout the surgery. The virus microinjection assembly consisted of a pulled glass micropipette (∼20 μm tip diameter pulled using a pipette puller) attached to a 1 ml syringe via plastic tubing. Micropipettes were filled with solution of virus using capillary force. The tip of the micropipette was gently inserted into the nodose ganglia. Virus (∼0.71 μl volume preintroduced into the pipette) was then injected by depressing the plunger (∼0.5 psi). For coinjection of AAV9-GFP and AAV9-Flex-tdT, two different viruses were premixed in 1:1 ratio. Only the left nodose ganglia received an injection. After AAV injection, atipamezole (5 mg/kg via subcutaneous injection) was used for rapid recovery. The animals were injected with meloxicam (500 mg/kg, s.c.) as a postanalgesic on the day and 24 h later. Four weeks later, mice were killed to collect vagal ganglia and brainstem.

### Intratracheal instillation of retrograde AAV for lung-specific nerve tracing

The AAV packaging plasmid vector pAAV-CAG-flex-tdTomato-WPRE was purchased from Addgene (catalog #51503) and incorporated into retrograde AAV2 by Boston Children’s Hospital Vector Core (rAAV-flex-tdT; 1.5 × 10^13^ GC/ml). Mice were anesthetized with a mixture of ketamine (50 mg/kg) and dexmedetomide (0.5 mg/kg) via intraperitoneal injection. For tracheal instillation, 30 μl of viral stock was diluted with 20 μl of Invitrogen Minimum Essential Medium (MEM; Thermo Fisher Scientific) for lung instillation via endotracheal intubation. The mouse was placed on a vertical stand, and the tongue was gently pulled to find the intubation path. An otoscope attached with a speculum was introduced to the larynx, and the vocal cords were identified. A 20 gauge intravenous catheter (1.5 inch; BD Insyte) was inserted. The otoscope was then retracted, and endotracheal intubation was confirmed by attaching a 1 ml syringe with a small amount of water in the tip: with proper endotracheal intubation, the liquid level in the syringe moves with respiration. The syringe was removed and 50 μl of virus/MEM mixture was pipetted into the catheter. The lung was then inflated with a 1 ml syringe filled with 300 μl of air to ensure instillation of the entire volume of the virus/MEM mixture. After instillation, atipamezole (5 mg/kg, s.c.) was used for rapid recovery. Four weeks later, mice were killed to collect vagal ganglia and medulla.

### Tissue collection and immunofluorescence

Mice were killed by CO_2_ inhalation and transcardially perfused with ice-cold PBS followed by perfusion fixation with ice-cold 3.7% formaldehyde (FA). Vagal ganglia and brainstem were dissected out and postfixed for 1 and 4 h, respectively, in 3.7% FA at 4°C. Tissue were washed in PBS to remove residual FA and transferred to 20% sucrose solution for cryoprotection. Tissue were mounted in OCT (optimal cutting temperature) compound and snap frozen in dry ice. Vagal ganglia were sectioned in 20 μm slices. The medulla was sectioned in either 30 or 40 μm slices. All slices were collected onto SuperFrost Plus slides. Slides were then air dried at room temperature in the dark overnight. For immunofluorescence, tissue was permeabilized with 0.3% Triton X-100 in PBS (PBSTx) for 15 min followed by blocking with 1% bovine serum albumin (BSA)/10% donkey serum (DS)/0.3% PBSTx. Tissue were incubated with primary antibodies diluted in blocking buffer overnight at 4°C. After washing with 0.2% Tween 20 in PBS (PBST) three times for 10 min, tissue was incubated with secondary antibodies in 1% BSA/5% DS in 0.2% PBST for 1 h. Tissue was washed with 0.2% PBST three times for 10 min and rinsed briefly with H_2_O. Slides were air dried and mounted with DPX Mounting Medium (Sigma-Aldrich).

### Vagal ganglia imaging and quantification

Vagal ganglia were stained for immunoreactivity to TRPV1 (goat; 1:150; catalog #sc-12498, Santa Cruz Biotechnology), and the neurotrophin receptors tyrosine receptor kinase A (TRKA; rabbit; 1:300; catalog #06–574, Millipore) and tyrosine receptor kinase B (TRKB; goat; 1:300; catalog #AF1494, R&D Systems). Primary antibodies were visualized with the following secondary antibodies: chicken anti-goat 647 (1:300; catalog #A212345, Invitrogen) and donkey anti-rabbit 488 (1:300; catalog #A21206, Invitrogen). Images were taken with Olympus FV1200 laser-scanning confocal microscope equipped with 20× UPLAN SAPO, 0.75 numerical aperture. *z*-Stack images (each 20 μm) of four to six different ganglia were taken, and projection images were obtained using Fiji software. Cell counts and somal diameters were measured using Fiji software. Each somal diameter was calculated as the mean of the longest and shortest distances across the soma of a neuron identified in a *z*-stack projection (2D image). The diameters of various vagal afferent subpopulations were compared using ANOVA with Sidak’s multiple-comparisons test (GraphPad Prism version 7). A *p* value <0.05 was considered significant.

### Medulla tissue clearing: passive CLARITY technique

Clearing of the medulla was performed using a modified version of the PACT (passive CLARITY technique; Treweek et al., 2015). Fixed medulla was submerged in prechilled hydrogel solution containing a 4% acrylamide (Bio-Rad) solution, a 0.25% thermal initiator (catalog #VA-044, Wako Chemicals) in PBS at 4°C for 3 d. Air bubbles were removed by vacuum on ice for 10 min, followed by a gentle nitrogen gas treatment for 10 min on ice. After heat activation of hydrogel polymerization by incubating medulla at 37°C for 3 h, medulla was transferred to 8% SDS (Sigma-Aldrich) after removing residual hydrogel solution for delipidation and incubated at 37°C for 5–7 d until tissue reach the desired transparency. Tissue was washed in 0.1% PBSTx for 24 h and washed again with PBS for 24 h at room temperature. For imaging, tissue was submerged in refractive index matching solution (sRIMS; 70% sorbitol in PBS; Sigma-Aldrich) for 24 h. The cleared medulla was mounted in sRIMS using an aluminum spacer between slide glass and coverslip. Images were taken and stitched using multiarea time-lapse imaging with an Olympus FV1200 laser-scanning confocal microscope. 3D volume images were processed with Imaris software (Oxford Instruments).

### Sequential imaging of the medulla

Medulla sections of 30 or 40 μm thickness were initially imaged with Nikon Eclipse microscope at 2× magnification (Nikon Elements). To aid comparison of reporter expression in the medulla, sections (of similar size) at the same rostral–caudal position were digitally overlaid in Adobe Photoshop CS5. In some cases, sequential images of the medulla were also used to reconstruct the 3D volume in TissueMaker software (MBF Bioscience). Regions of interest (e.g., reporter expression) were contoured using Tissue Mapper software (MBF Bioscience) prior to visualization. For higher-magnification visualization of the medulla, 40 μm sequential brain sections were counterstained with either green or blue fluorescent Invitrogen Nissl staining (1:600; NeuroTrace 500/525 or 435/455, Fluorescent Nissl Stain, Thermo Fisher Scientific). In addition, 40 μm tissue sections of TRPV1-tdT and Tac1-tdT strains were immunostained with anti-TRPV1 (guinea pig; 1:150 at room temperature; catalog #gp14100, Neuromics) followed by secondary antibody incubation with donkey anti-guinea pig 647 (1:300; catalog #AP193SA6, Millipore). To increase the AAV-mediated reporter signal in central terminations, 40 μm tissue sections were immunostained with rabbit anti-DsRed (1:300; catalog #632496, Clontech) and chicken anti-GFP (1:1000; catalog #ab13970, Abcam). For secondary antibodies, either goat anti-chicken 647 (1:300; catalog #ab150171, Abcam) or Invitrogen goat anti-chicken 488 (1:300; catalog #A32931, Thermo Fisher Scientific) in combination with Invitrogen donkey anti-rabbit 546 (1:300; catalog #A10040, Thermo Fisher Scientific). In the cases where the α-GFP immunoreactivity was visualized using the secondary antibody with goat anti-chicken 647, both the native GFP and α-GFP immunoreactivity images were pseudocolored to green for presentation purposes. Stained tissue was imaged (*z*-stack images, each of 40 μm) with an Andor Dragonfly spinning disk confocal microscope using Fusion software, and projection images were processed with Imaris software. For the composite image of Tac1^+^ afferents innervating the lungs, *z*-stacks of four consecutive coronal nTS sections were used. Each *z*-stack was divided into two separate projections (top and bottom) and pseudocolored to a distinct color in the rainbow. All eight projections were then aligned in Adobe Photoshop CS5 software. In all cases, the identification of anatomic structures and subnuclei were based on the mouse brain map (Paxinos and Franklin, 2012).

## Results

### Characterization of reporter expression in the vagal ganglia

We investigated tdT expression in the vagal ganglia of offspring from crosses of ROSA26-loxP-STOP-loxP-tdTomato mice with either Pirt-Cre, 5-HT3-Cre, TRPV1-Cre, or Tac1-Cre mice. The resultant Pirt-tdT, 5-HT3-tdT, TRPV1-tdT, and Tac1-tdT mice grew normally and had no obvious pathophysiological phenotype. Robust tdTomato expression was seen in neuronal soma and axons in vagal ganglia in all mice, although different neuronal subsets were labeled in the different strains.

**Figure 1.**
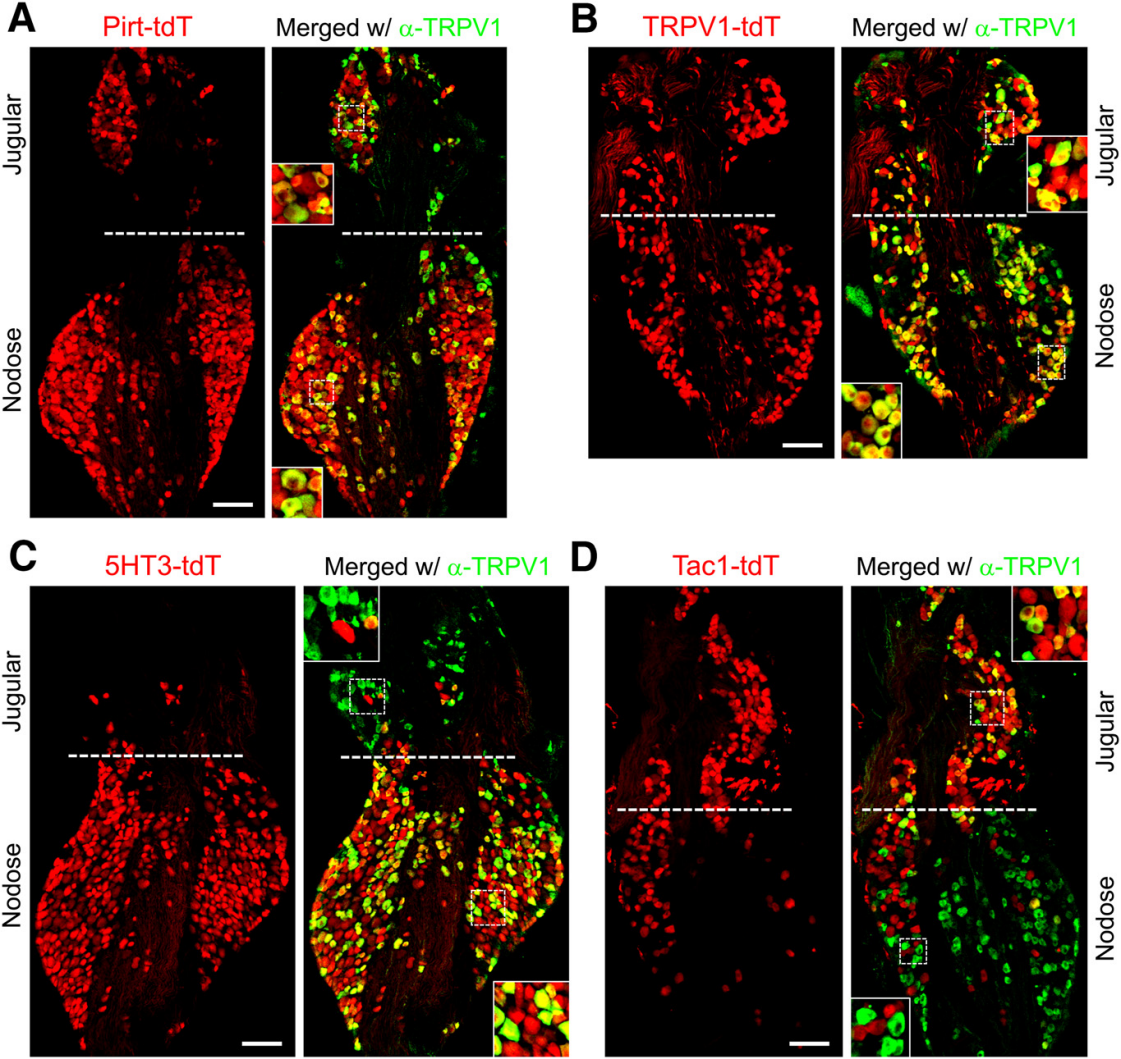
tdTomato expression and α-TRPV1 immunoreactivity in vagal ganglia. ***A***, Pirt-tdT. ***B***, TRPV1-tdT. ***C***, 5-HT3-tdT. ***D***, Tac1-tdT. Native tdTomato expression (red) is shown on the left, with overlap with α-TRPV1 immunoreactivity on the right (green). Scale bar, 100 μm; insets show enlarged views of both jugular and nodose neurons. Data are representative of *n* = 3 animals for each strain.

**Figure 2.**
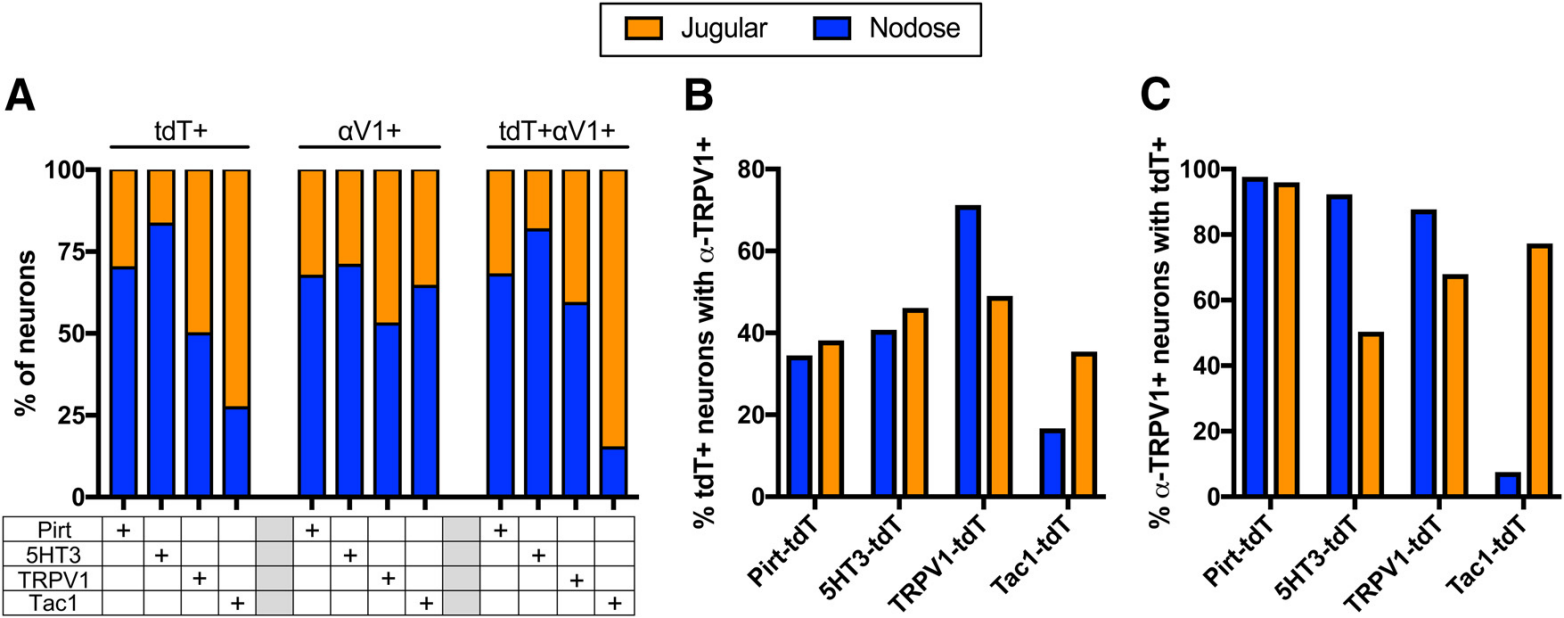
Comparison of tdTomato expression and α-TRPV1 immunoreactivity in vagal ganglia of Pirt-tdT, 5-HT3-tdT, TRPV1-tdT, and Tac1-tdT. ***A***, The relative contribution of jugular (orange bars) or nodose (blue bars) neurons to the vagal neuronal populations expressing tdTomato (tdT^+^), α-TRPV1 immunoreactivity (αV1^+^), or both tdTomato and α-TRPV1 immunoreactivity (tdT^+^αV1^+^). ***B***, The percentage of tdTomato-expressing neurons in the nodose (blue) or jugular (orange) ganglia with α-TRPV1 immunoreactivity. ***C***, The percentage of α-TRPV1-immunoreactive neurons in the nodose (blue) or jugular (orange) ganglia with tdTomato expression. Data are derived from *n* = 3 animals in each strain.

In the Pirt-tdT mice, tdTomato expression was widespread throughout both the nodose and jugular ganglia, and this included both TRPV1-expressing neurons (determined by immunofluorescence) and TRPV1^−^ neurons (Figs. 1*A*, 2). Thirty-five to forty percent of tdTomato^+^ neurons were labeled by α-TRPV1 immunoreactivity in both the nodose and jugular ganglia (Fig. 2*B*). More than 95% of nodose and jugular neurons with α-TRPV1 immunoreactivity expressed tdTomato in Pirt-tdTomato ganglia (Fig. 2*C*). In the TRPV1-tdT mice, tdTomato expression was observed in a subset of nodose and jugular neurons (Fig. 1*B*). Interestingly, not all tdTomato-expressing neurons had α-TRPV1 immunoreactivity (Figs. 1*B*, 2*B*). Offspring from crosses of ROSA26-loxP-STOP-loxP-tdTomato mice with Cre-expressing mice have permanent reporter expression in cells that express the gene-specific Cre. As such, transient embryological expression of the gene-specific Cre causes reporter expression regardless of gene expression in the adult. Our data suggest that some vagal neurons only have transient expression of TRPV1 during development. In the 5-HT3-tdT mice, tdTomato expression was largely restricted to nodose neurons (Figs. 1*C*, 2*A*): the ratio of nodose to jugular neurons expressing tdTomato was 5:1, compared with 2.3:1 and 1:1 for Pirt-tdT and TRPV1-tdT, respectively. Forty percent of 5-HT3-tdT^+^ neurons were also labeled by α-TRPV1 immunoreactivity (Fig. 2*B*). In Tac1-tdT ganglia, tdTomato expression was observed in many jugular neurons and in a few nodose neurons (Figs. 1*D*, 2*A*). Compared with the jugular neurons, where tdTomato expression often overlapped with α-TRPV1 immunoreactivity, very few nodose tdTomato^+^ neurons had α-TRPV1 immunoreactivity (Figs. 1*D*, 2*B*,*C*). As such, the nodose/jugular ratio for neurons coexpressing tdTomato and α-TRPV1 immunoreactivity was 0.18:1.

**Figure 3.**
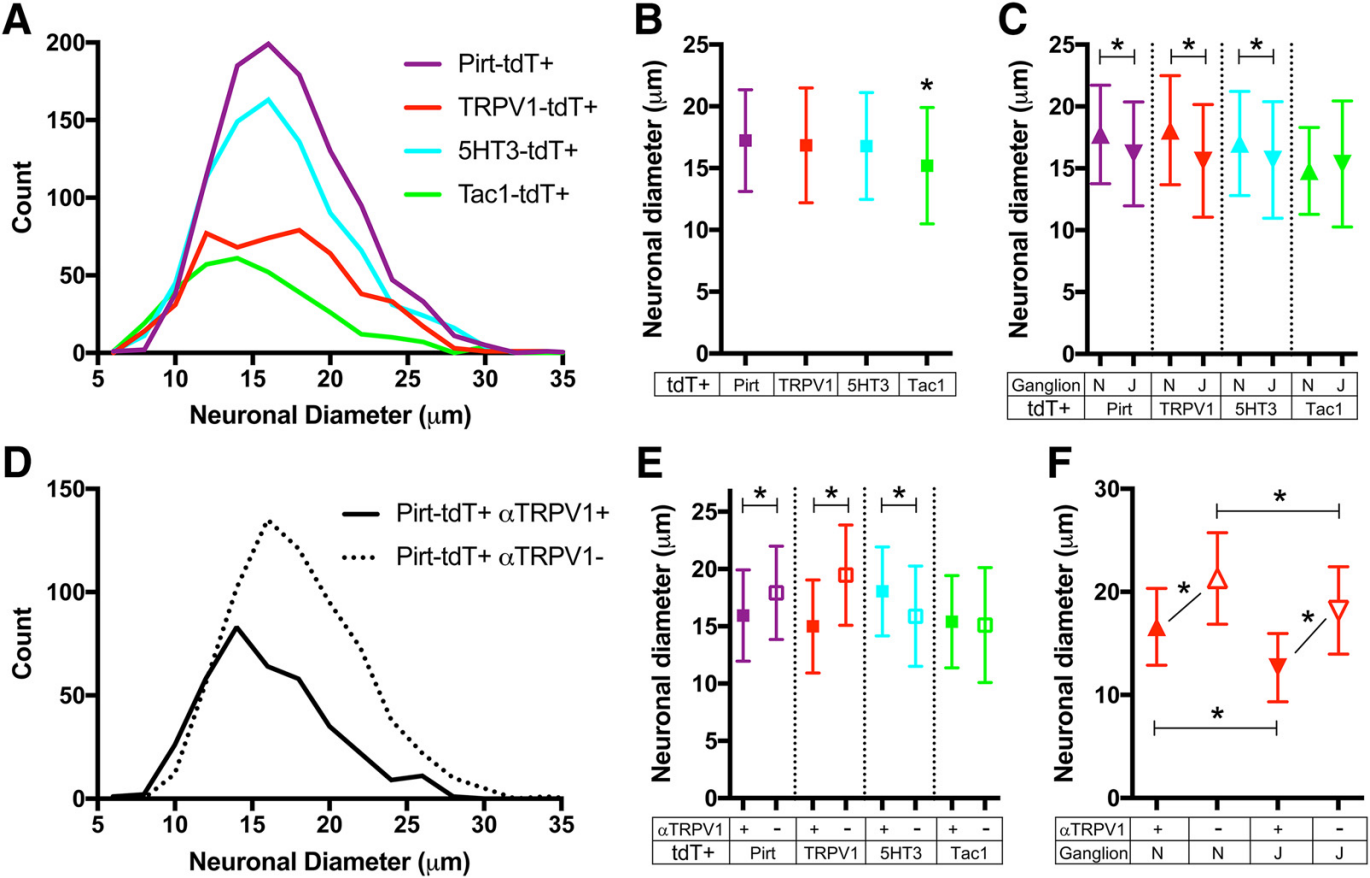
Neuronal diameters of tdTomato^+^ vagal neurons from Pirt-tdT, 5-HT3-tdT, TRPV1-tdT, and Tac1-tdT. ***A***, Histogram of neuronal diameter of tdTomato^+^ vagal neurons from Pirt-tdT (*n* = 1040 neurons), 5-HT3-tdT (*n* = 850 neurons), TRPV1-tdT (*n* = 501 neurons), and Tac1-tdT (*n* = 330 neurons). ***B***, Mean ± SD neuronal diameter of tdTomato^+^ vagal neurons from Pirt-tdT, 5-HT3-tdT, TRPV1-tdT, and Tac1-tdT. ***C***, Mean ± SD neuronal diameter of tdTomato^+^ nodose neurons (upward triangles) compared with jugular neurons (downward triangles) from Pirt-tdT, 5-HT3-tdT, TRPV1-tdT, and Tac1-tdT. ***D***, Histogram of neuronal diameter of tdTomato-expressing neurons with α-TRPV1 immunoreactivity (Pirt-tdT^+^αTRPV1^+^, black line) and tdTomato-expressing neurons without α-TRPV1 immunoreactivity (Pirt-tdT^+^αTRPV1^−^, dotted line) from Pirt-tdT. ***E***, Mean ± SD neuronal diameter of tdTomato^+^ vagal neurons with (filled squares) and without (open squares) α-TRPV1 immunoreactivity from Pirt-tdT, 5-HT3-tdT, TRPV1-tdT, and Tac1-tdT. ***F***, Mean ± SD neuronal diameter of tdTomato^+^ nodose (upward triangles) and jugular (downward triangles) neurons with (filled squares) and without (open squares) α-TRPV1 immunoreactivity from TRPV1-tdT. *Denotes significant difference (*p* < 0.05, ANOVA with Sidak’s multiple comparisons).

We measured the somal diameter of tdTomato-expressing vagal neurons. In general, there was substantial overlap between the labeled subsets, ranging from ∼7–30 μm (Fig. 3*A*). Tac1-tdT^+^ vagal neurons were smaller than tdT^+^ neurons from the other three strains (*p* < 0.05; Fig. 3*A*,*B*). The nodose populations of Pirt-tdT^+^, 5-HT3-tdT^+^, and TRPV1-tdT^+^ neurons were larger than their jugular counterparts (*p* < 0.05; Fig. 3*C*), although this was not the case for Tac1-tdT^+^ neurons. Consistent with previous reports that nociceptive neurons are smaller than non-nociceptive neurons (Lawson et al., 1993; Zhuo et al., 1997), we found that Pirt-tdT^+^ neurons with α-TRPV1 immunoreactivity were smaller than Pirt-tdT^+^ neurons lacking α-TRPV1 immunoreactivity (*p* < 0.05; Fig. 3*D*,*E*). This was also true for TRPV1-tdT^+^ neurons (*p* < 0.05) but not for Tac1-tdT^+^ neurons (*p* > 0.05; Fig. 3*E*). Surprisingly, 5-HT3-tdT^+^ neurons with α-TRPV1 immunoreactivity were larger than 5-HT3-tdT^+^ neurons lacking α-TRPV1 immunoreactivity (*p* < 0.05; Fig. 3*E*). The negative correlation of α-TRPV1 immunoreactivity on somal size was observed in both nodose and jugular TRPV1-tdT^+^ neurons (*p* <0.05; Fig. 3*F*).

**Figure 4.**
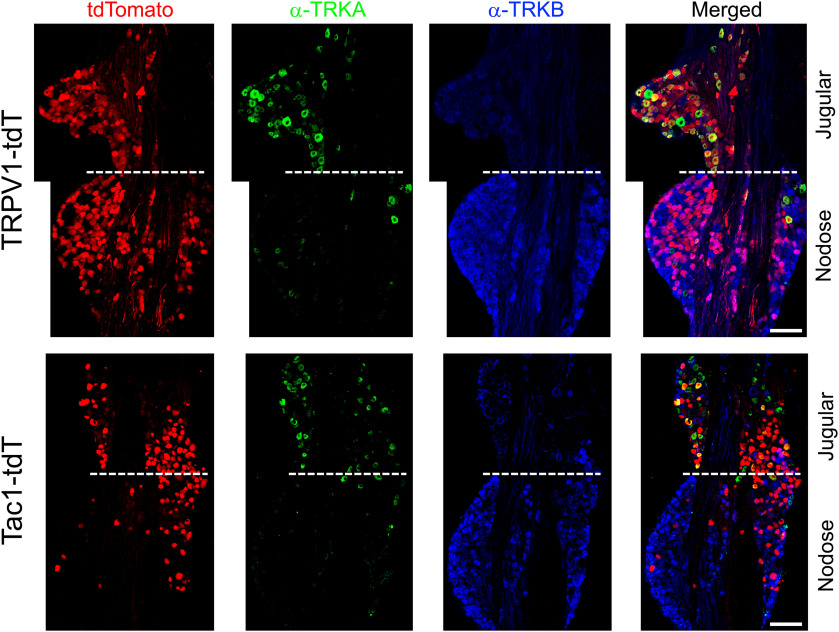
Native tdTomato expression, α-TRKA immunoreactivity and α-TRKB immunoreactivity in vagal ganglia. Top, TRPV1-tdT. Bottom, Tac1-tdT. Scale denotes 100 μm. Data are representative of *n* = 3 animals for each strain.

**Figure 5.**
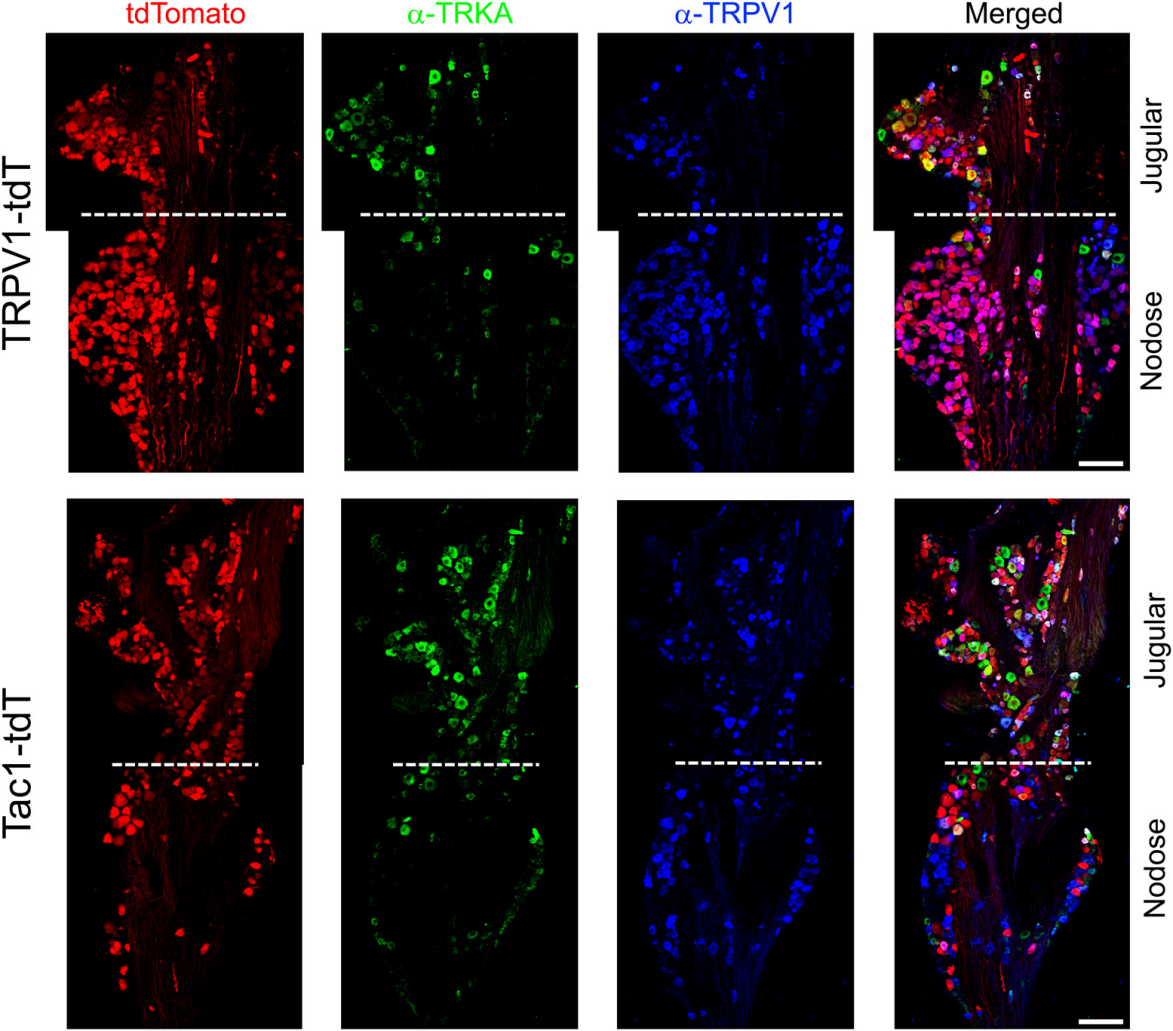
Native tdTomato expression, α-TRKA immunoreactivity, and α-TRPV1 immunoreactivity in vagal ganglia. Top, TRPV1-tdT. Bottom, Tac1-tdT. Scale bar, 100 μm. Data are representative of *n* = 3 animals for each strain.

**Figure 6.**
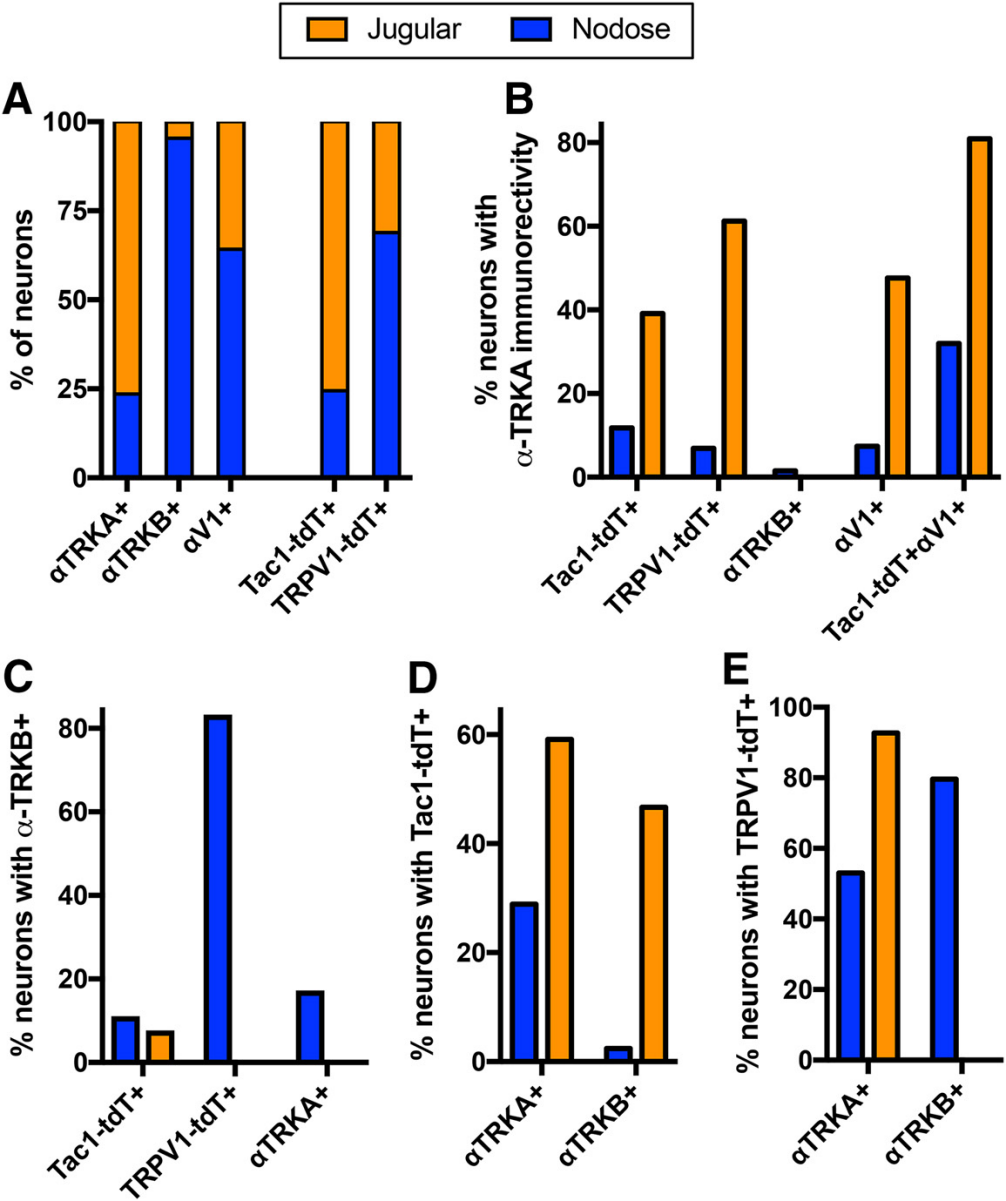
Comparison of tdTomato expression, α-TRPV1 immunoreactivity, α-TRKA immunoreactivity, and α-TRKB immunoreactivity in vagal ganglia of TRPV1-tdT and Tac1-tdT. ***A***, The relative contribution of jugular (orange bars) or nodose (blue bars) neurons to the vagal neuronal populations expressing α-TRKA immunoreactivity (αTRKA^+^), α-TRKB immunoreactivity (αTRKB^+^), α-TRPV1 immunoreactivity (αV1^+^), or tdTomato in either the Tac1-tdT or the TRPV1-tdT. ***B***, The percentage of specific neuronal groups in the nodose (blue) or jugular (orange) ganglia with α-TRKA immunoreactivity. ***C***, The percentage of specific neuronal groups in the nodose (blue) or jugular (orange) ganglia with α-TRKB immunoreactivity. ***D***, The percentage of neurons expressing either α-TRKA immunoreactivity (αTRKA^+^) or α-TRKB immunoreactivity (αTRKB^+^) in the nodose (blue) or jugular (orange) ganglia with tdTomato expression in the Tac1-tdT ganglia. ***E***, The percentage of neurons expressing either α-TRKA immunoreactivity (αTRKA^+^) or α-TRKB immunoreactivity (αTRKB^+^) in the nodose (blue) or jugular (orange) ganglia with tdTomato expression in the TRPV1-tdT ganglia. Data are derived from *n* = 3 animals in each strain.

Because of their distinct embryological sources, the nodose and jugular ganglia have different neurotrophin receptor expression (Nassenstein et al., 2010; Lieu et al., 2011; Wang et al., 2017; Kupari et al., 2019). We investigated the expression of TRKA and TRKB in the vagal TRPV1-tdT^+^ and Tac1-tdT^+^ populations using immunofluorescence labeling. α-TRKA immunoreactivity was observed in many jugular neurons and in very few nodose neurons (Figs. 4, 5, 6*A*). Whereas α-TRKB immunoreactivity was almost exclusively observed in nodose neurons (Figs. 4, 6*A*). There was very little overlap between α-TRKA immunoreactivity and α-TRKB immunoreactivity (Figs. 4, 6*B*,*C*). Sixty-one percent of jugular TRPV1-tdT^+^ neurons had α-TRKA immunoreactivity, whereas this was <7% in the nodose ganglia (Figs. 4, 5, 6*B*). Eighty-three percent of nodose TRPV1-tdT^+^ neurons had α-TRKB immunoreactivity, whereas this was <1% in the jugular ganglia (Figs. 4, 6*C*). Although 39% of jugular Tac1-tdT^+^ neurons had α-TRKA immunoreactivity, this correlation of Tac1 and TRKA expression was much higher in the jugular Tac1-tdT^+^ neurons which also coexpressed α-TRPV1 immunoreactivity (81%; Figs. 4, 5, 6*B*). Less than 8% of Tac1-tdT^+^ neurons had α-TRKB immunoreactivity. Consistent with the widespread expression of Tac1 in jugular neurons, 59% of jugular neurons with α-TRKA immunoreactivity were Tac1-tdT^+^ (Fig. 6*D*). Whereas, TRPV1-tdT^+^ expression was observed in >90% of jugular neurons with α-TRKA immunoreactivity and in 80% of nodose neurons with α-TRKB immunoreactivity (Fig. 6*E*).

### Characterization of reporter expression in the medulla

**Figure 7.**
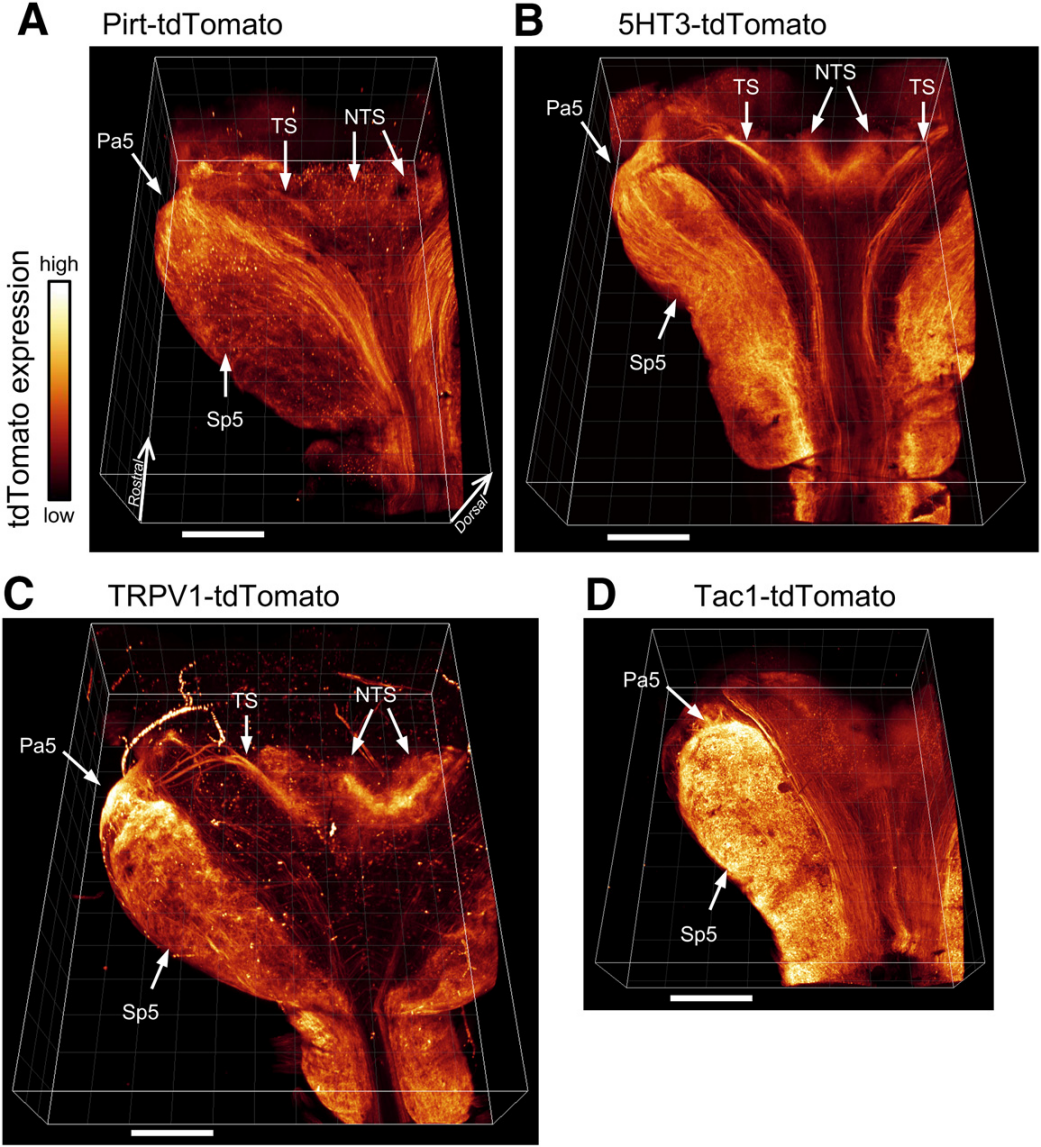
Native tdTomato expression in cleared whole-mount medulla. ***A***, Pirt-tdT. ***B***, 5-HT3-tdT. ***C***, TRPV1-tdT. ***D***, Tac1-tdT. All medulla are displayed in the same dorsal aspect orientation. The following structures are identified: nTS, Pa5, Sp5, and TS. Scale bar, 1 mm.

The vagus nerve provides afferent signaling to brainstem networks in the medulla. To identify the central projections of the specific afferent subsets labeled in the Pirt-tdT, 5-HT3-tdT, TRPV1-tdT, and Tac1-tdT mice, we first performed whole-mount imaging of cleared medulla (Fig. 7). Widespread tdTomato expression was observed in both the nTS (central terminations of the facial nerve, glossopharyngeal nerve, and vagal nerve) and the spinal trigeminal nucleus (Sp5; central terminations of the trigeminal nerve). Strong tdTomato expression was also noted in the paratrigeminal complex (Pa5). tdTomato expression in the TS was particularly evident in the 5-HT3-tdT and TRPV1-tdT strains (Fig. 7*B*,*C*), with between three and five reporter-labeled branches entering the ventral aspect of the lateral wall and proceeding medially at a slight rostral angle, before grossly coalescing into a single bundle and heading caudally and medially into the nTS. Multiple afferent tractus solitarius “rootlets” in the lateral wall of the medulla have previously been reported in the rat (Kalia and Sullivan, 1982). Importantly, tdTomato expression was observed to a differing extent in all strains in some intrinsic medullary neurons. Indeed, tdTomato labeling of the nTS and TS in the Tac1-tdT strain was difficult to resolve in the whole-mount medulla.

**Figure 8.**
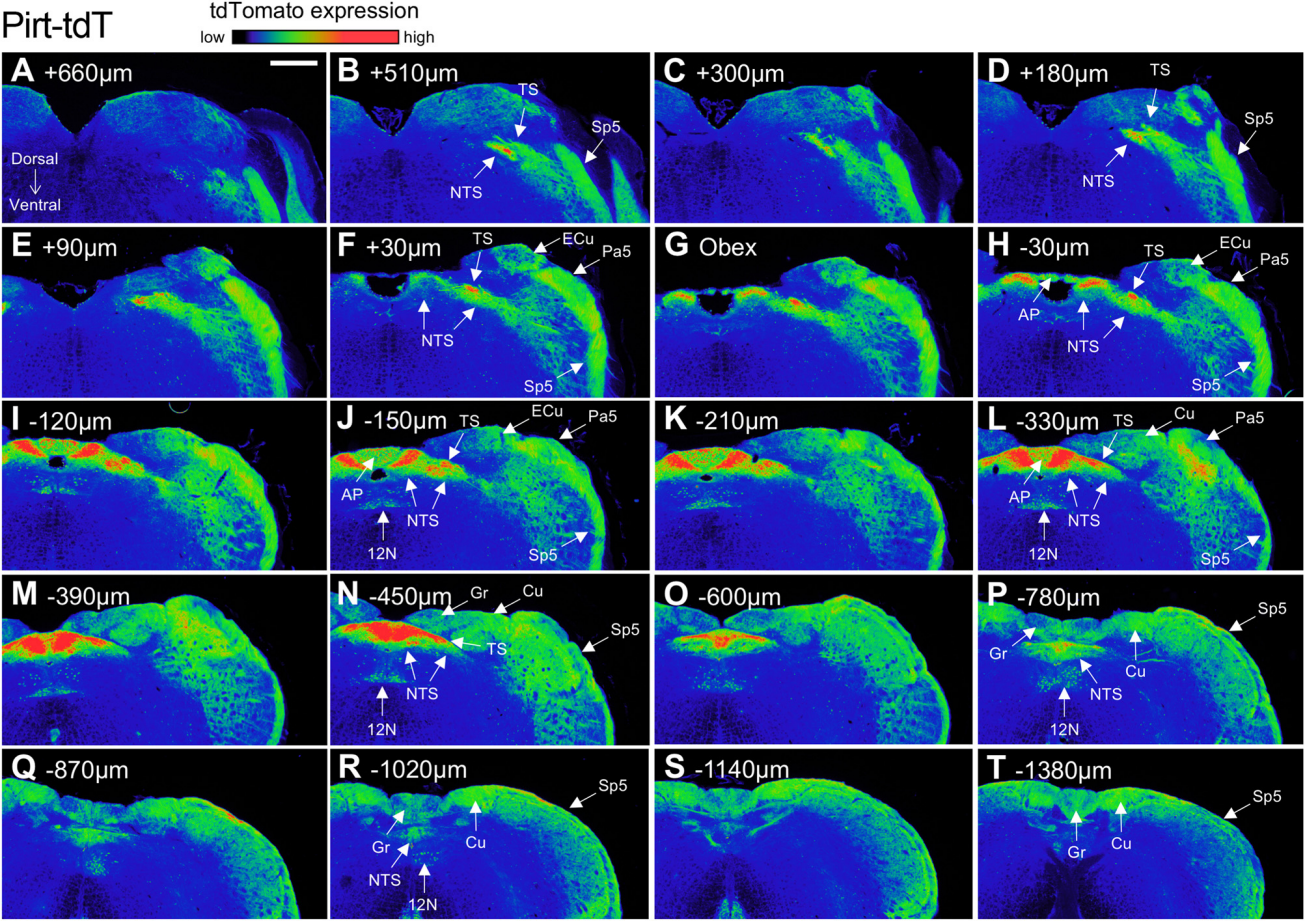
tdTomato expression in serial coronal sections of the medulla in Pirt-tdT. ***A–T***, Data presented from rostral to caudal, with labeling for the position relative to obex. The intensity of native tdTomato expression is shown in rainbow pseudocolor. The following structures are identified: area postrema (AP), cuneate nucleus (Cu), external cuneate nucleus (ECu), gracile nucleus (Gr), 12N, nTS, Pa5, Sp5, and TS. Scale bar, 400 μm. Data are representative of *n* = 8 animals.

**Figure 9.**
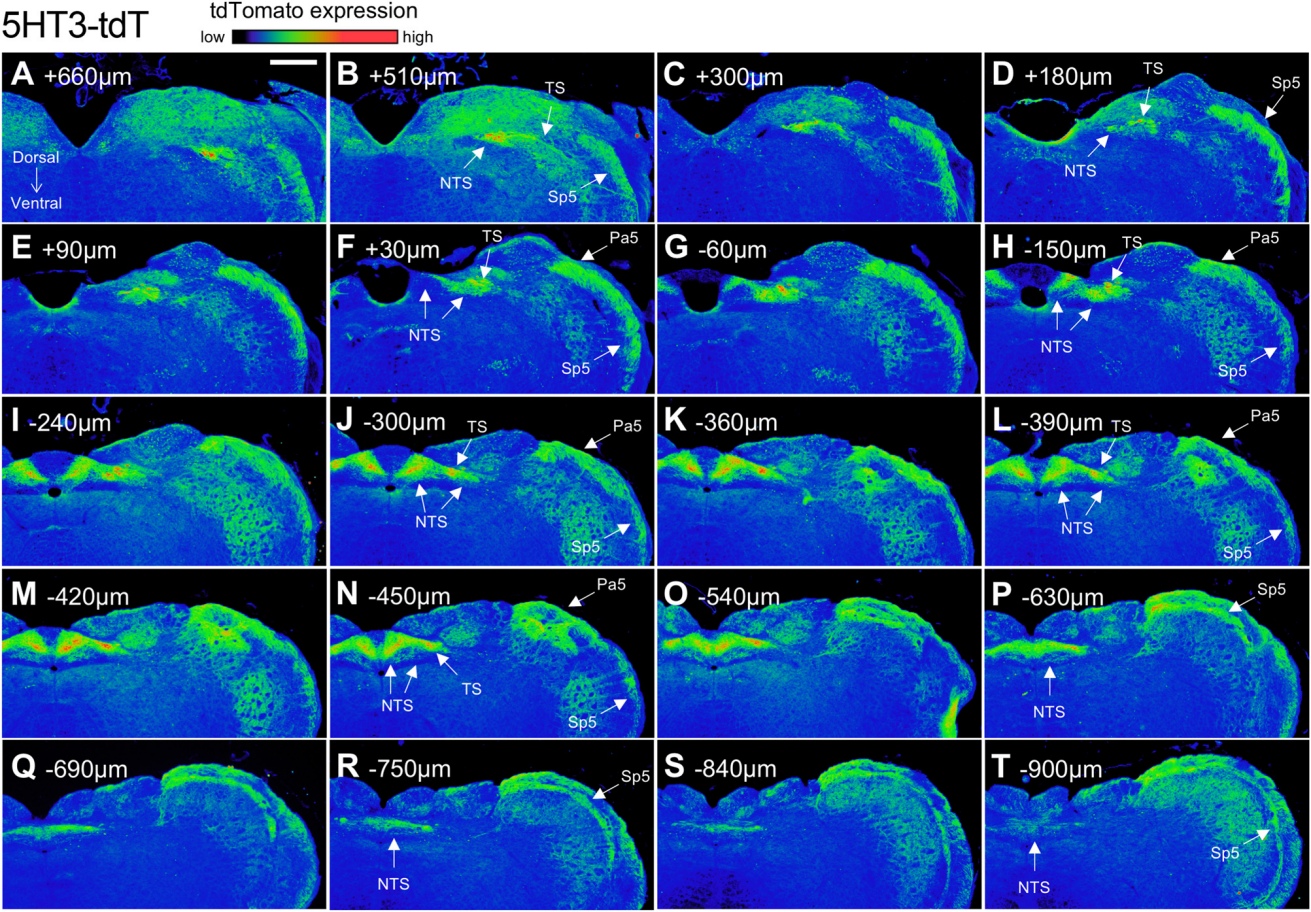
tdTomato expression in serial coronal sections of the medulla in 5-HT3-tdT. ***A–T***, Data presented from rostral to caudal, with labeling for the position relative to obex. The intensity of native tdTomato expression is shown in rainbow pseudocolor. The following structures are identified: nTS, Pa5, Sp5, and TS. Scale bar, 400 μm. Data are representative of *n* = 6 animals.

**Figure 10.**
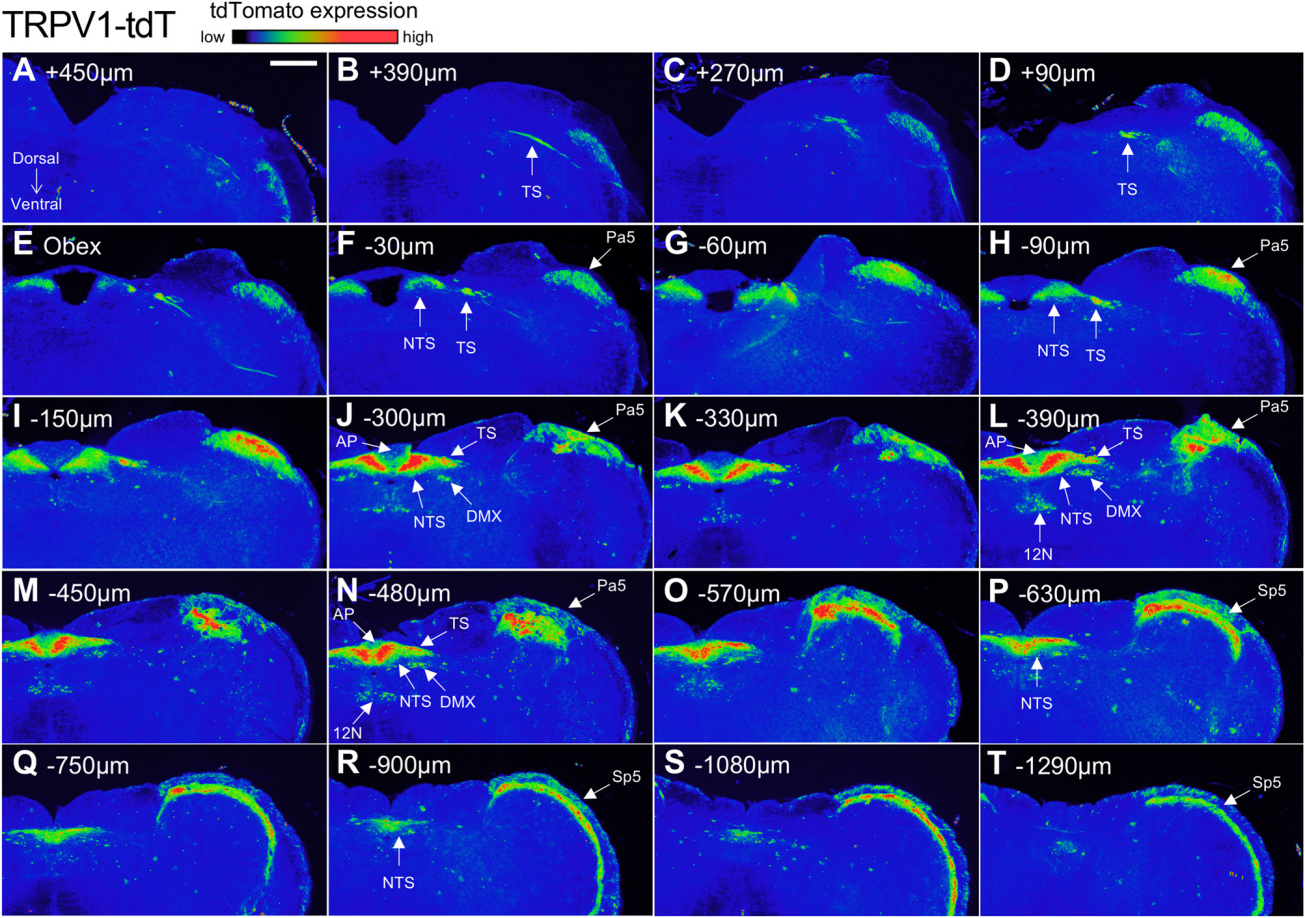
tdTomato expression in serial coronal sections of the medulla in TRPV1-tdT. ***A–T***, Data presented from rostral to caudal, with labeling for the position relative to obex. The intensity of native tdTomato expression is shown in rainbow pseudocolor. The following structures are identified: area postrema (AP), DMX, 12N, nTS, Pa5, Sp5, and TS. Scale bar, 400 μm. Data are representative of *n* = 6 animals.

**Figure 11.**
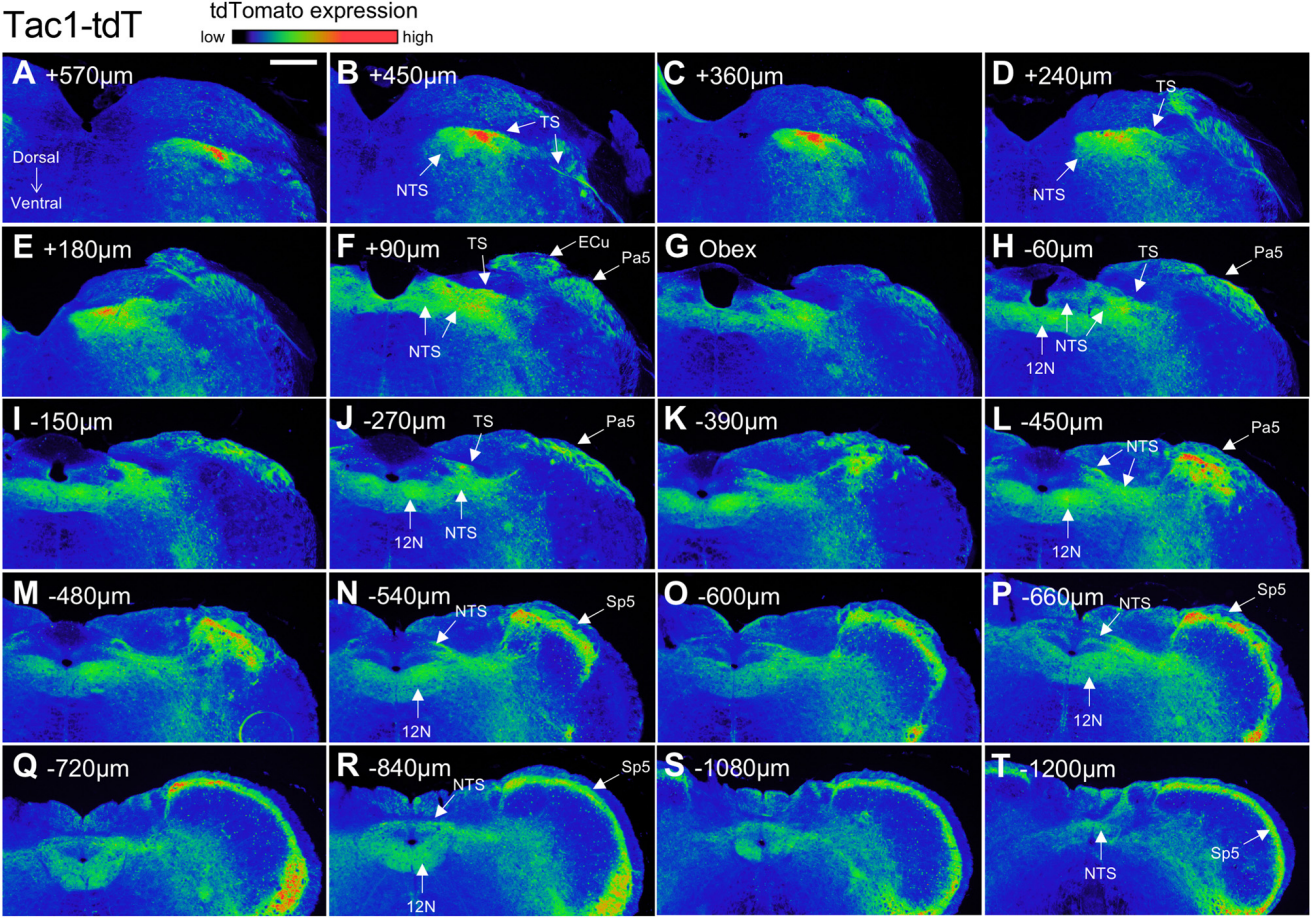
tdTomato expression in serial coronal sections of the medulla in Tac1-tdT. ***A–T***, Data presented from rostral to caudal, with labeling for the position relative to obex. The intensity of native tdTomato expression is shown in rainbow pseudocolor. The following structures are identified: external cuneate nucleus (ECu), 12N, nTS, Pa5, Sp5, and TS. Scale bar, 400 μm. Data are representative of *n* = 5 animals.

Serial sectioning of the medulla provided further detail of the tdTomato expression in the four strains (Figs. 8–11). At low magnification, reporter expression in the medulla of Pirt-tdT mice was shown to be largely restricted to areas associated with sensory pathways, including the TS, nTS, Sp5, Pa5, area postrema, external cuneate, cuneate, and gracile nucleus. The dense reporter signal in the nTS at this magnification prevented definitive determination of the contribution of fibers or neurons to the tdTomato expression. We did note, however, a limited number of intrinsic neurons within the hypoglossal and the caudal nucleus ambiguus (data not shown) were also labeled (Fig. 8). In the 5-HT3-tdT medulla, robust tdTomato expression was observed in the TS, nTS, Sp5, and Pa5, and to a lesser extent in the area postrema, external cuneate, cuneate, and gracile nucleus (Fig. 9). In addition, there was robust tdTomato expression in the medial vestibular nucleus. In the TRPV1-tdT mouse, tdTomato expression was observed in the tractus solitarius, nTS, Sp5, Pa5, and area postrema (Fig. 10). In addition, a subpopulation of intrinsic neurons within the dorsal motor nucleus of the vagus (DMX) and the hypoglossal nucleus (12N) were labeled with tdTomato. Compared with the Pirt-tdT and 5-HT3-tdT, tdTomato expression in the TRPV1-tdT medulla as was almost completely restricted to these areas—without the “background” signal from the occasional neuron/fiber expressing the reporter. Reporter expression in the Tac1-tdT medulla was widespread, although there were particularly high signals in the tractus solitarius, Sp5, Pa5, and the hypoglossal nucleus (Fig. 11).

**Figure 12.**
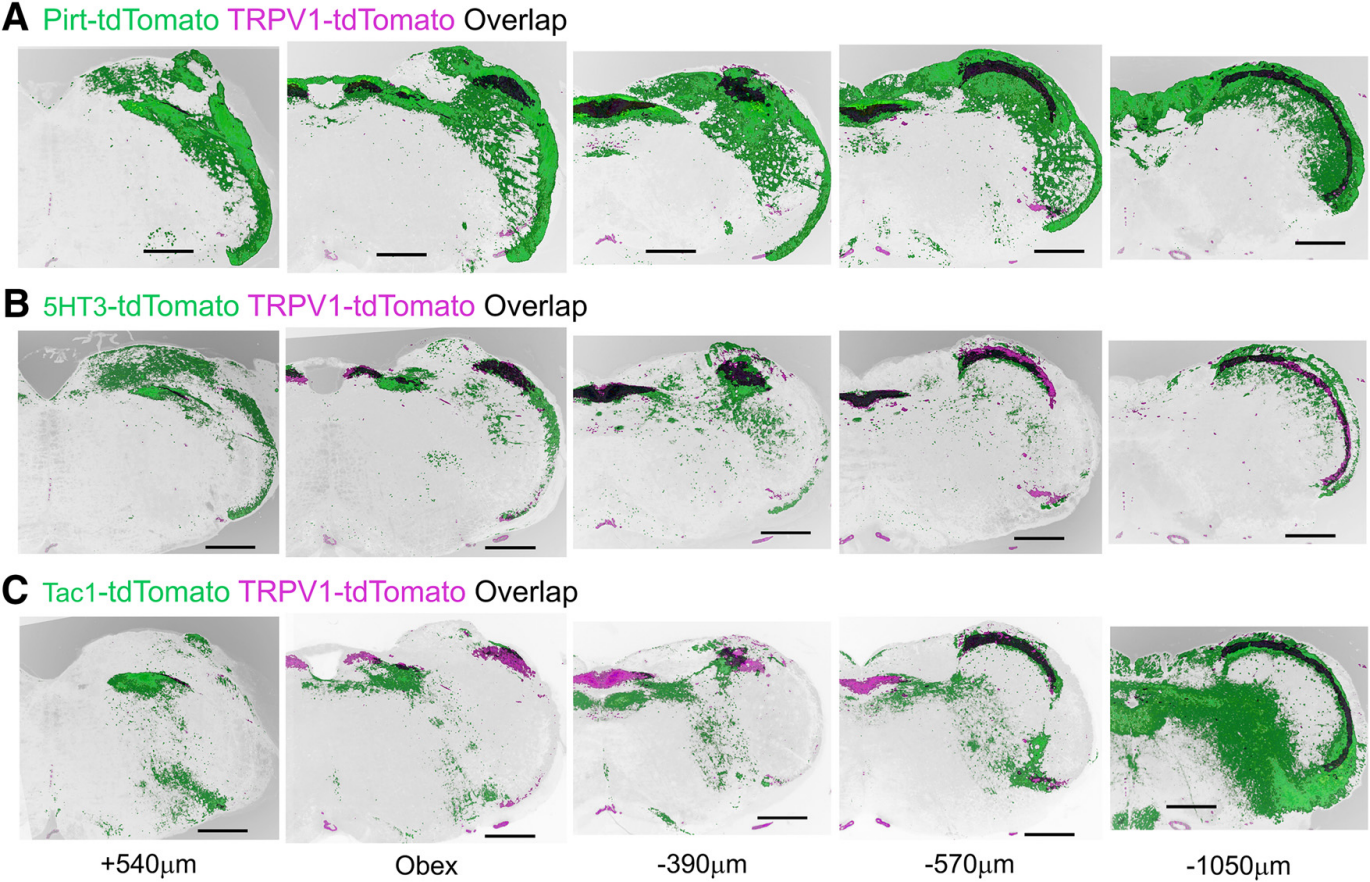
Comparison of native tdTomato expression in coronal sections of the medulla. ***A–C***, tdTomato expression in TRPV1-tdT (purple) is compared with tdTomato expression in Pirt-tdT (***A***; green), 5-HT3-tdT (***B***; green), and Tac1-tdT (***C***; green) and overlap of reporter expression is denoted in black. Data presented from rostral (left) to caudal (right), with labeling for the position relative to obex. Scale bar, 500 μm.

The nTS has a distinct cytoarchitecture, which has subdivided into specific subnuclei in multiple species (Kalia and Mesulam, 1980; Kalia and Sullivan, 1982; Kubin et al., 2006; Paxinos and Franklin, 2012). The images from select sections of medulla were digitally overlaid in order to determine the comparative tdTomato expression in the four strains (Fig. 12). tdTomato labeling was observed in the Pirt-tdT and 5-HT3-tdT strains throughout the lateral and medial nTS subnuclei, from rostral areas (+500 μm, relative to obex) through to the furthermost caudal areas (−1100 μm). Similarly, there was robust tdTomato expression in the Sp5 along the entire rostral–caudal axis of the medulla in these two strains. The major difference between the reporter expression in the Pirt-tdT and 5-HT3-tdT is the low expression in the area postrema, external cuneate, cuneate, and gracile nucleus in the 5-HT3-tdT compared with Pirt-tdT. In the TRPV1-tdT, reporter expression in the nTS is largely restricted to medial subnuclei, in particular in areas at the obex and more caudally. Similarly, the robust reporter labeling of the Sp5 occurs in caudal aspects of the medulla, with little in areas rostral to obex. Interestingly, given the overlap of Tac1-tdT expression and α-TRPV1 immunoreactivity in the vagal ganglia (Figs. 1, 2), there is little obvious overlap of reporter expression in the Tac1-tdT and TRPV1-tdT strains in the nTS (Fig. 12*C*). Nevertheless, there is substantial overlap of Tac1-tdT and TRPV1-tdT in the Pa5 and the caudal Sp5.

**Figure 13.**
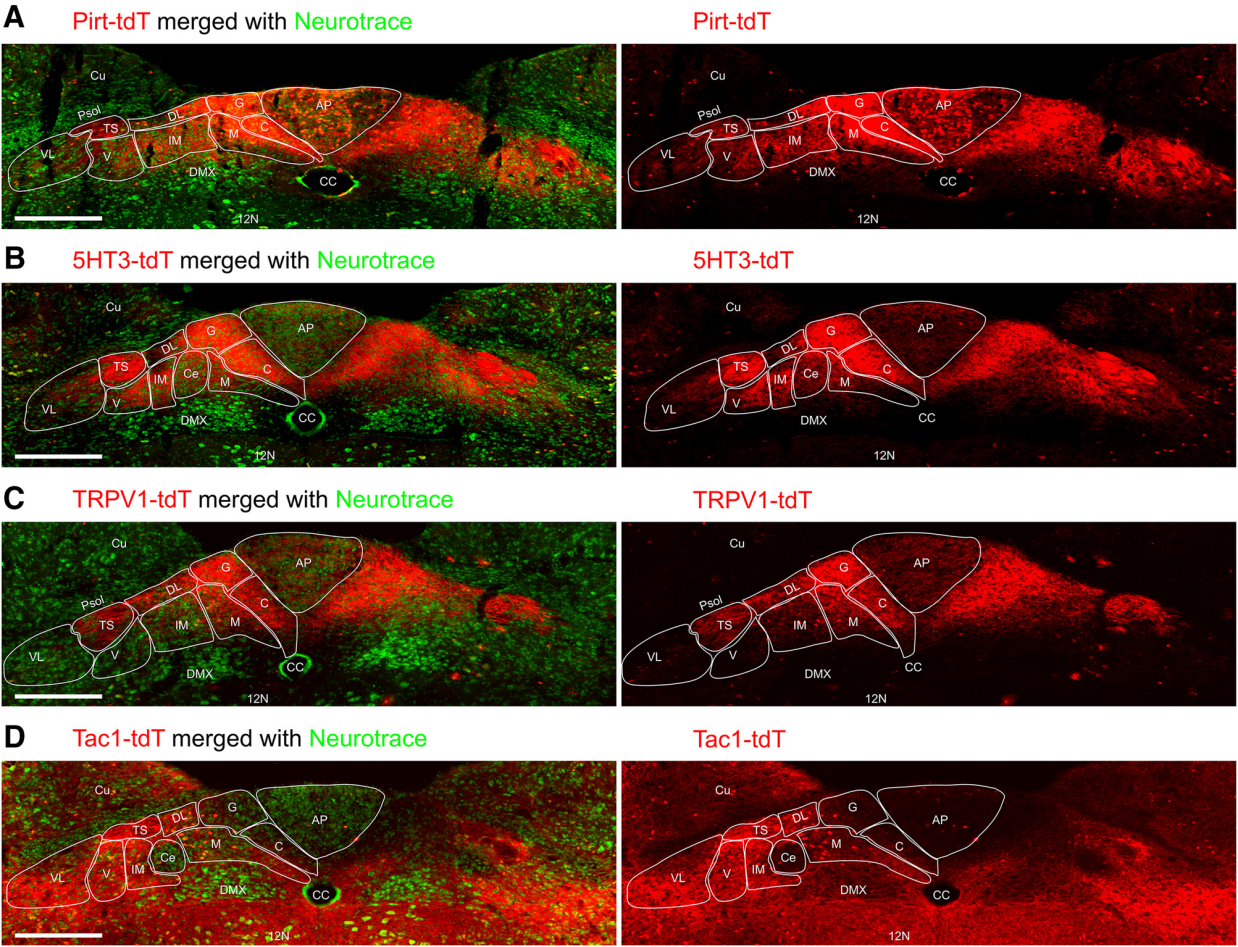
tdTomato expression in nTS subnuclei. ***A***, Pirt-tdT at −330 μm (relative to obex). ***B***, 5-HT3-tdT at −300 μm. ***C***, TRPV1-tdT at −360 μm. ***D***, Tac1-tdT at −330 μm. Left, Native tdTomato expression (red) merged with neurotrace labeling of intrinsic neurons (green). Right, tdTomato expression alone. The following structures are identified: area postrema (AP), central canal (CC), cuneate nucleus (Cu), DMX, 12N, parasolitary nucleus (Psol), SolC (C), SolCe (Ce), SolDL (DL), SolG (G), SolIM (IM), SolM (M), SolV (V), SolVL (VL), and TS. Scale bar, 300 μm.

**Figure 14.**
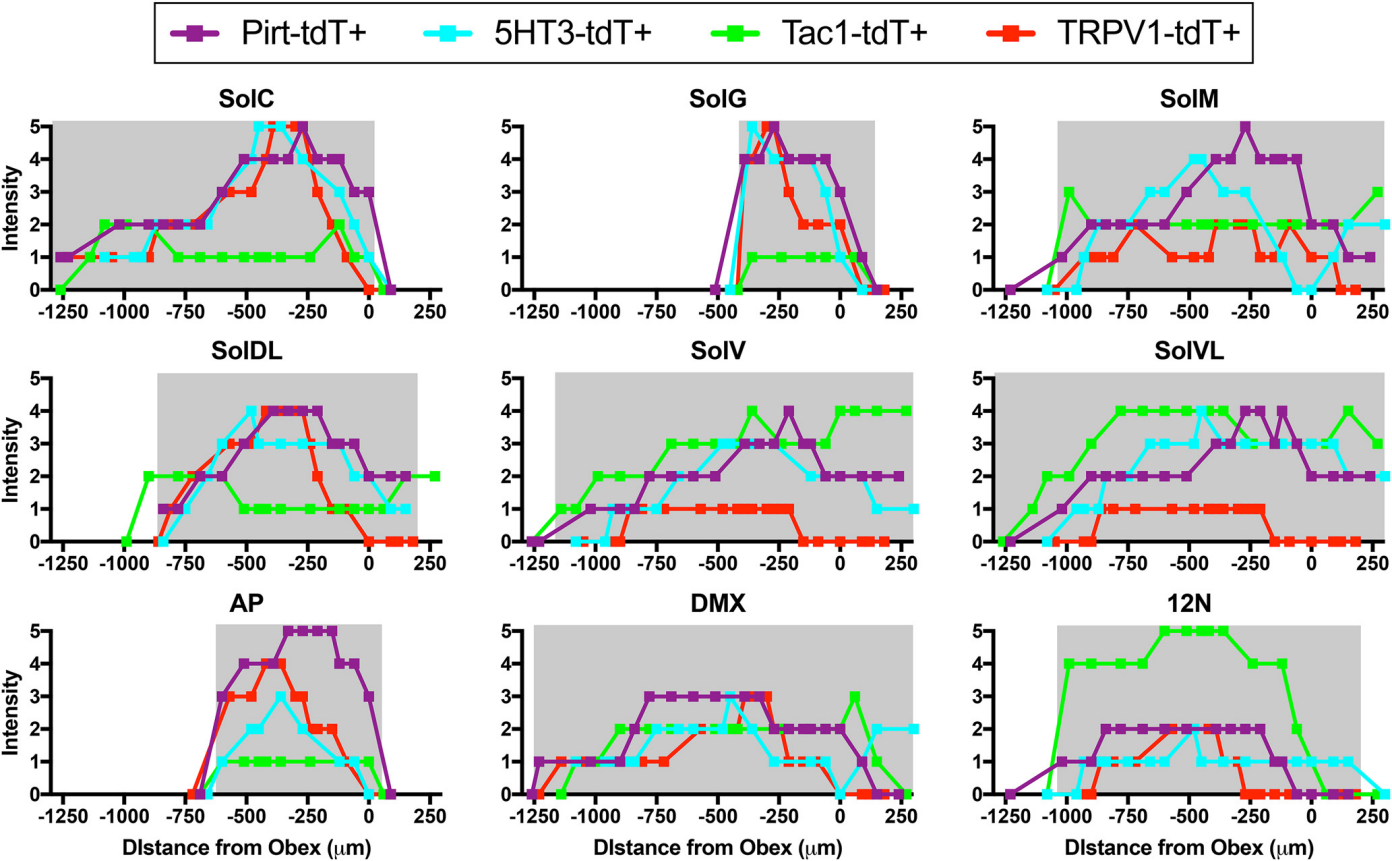
tdTomato expression in dorsal medulla subnuclei along complete rostral–caudal axis. Data are mean native tdTomato intensity in SolC, SolG, SolM, SolDL, SolV, SolVL, area postrema (AP), DMX, and 12N from Pirt-tdT (purple), 5-HT3-tdT (cyan), TRPV1-tdT (red), and Tac1-tdT (green; *n* = 5–8 mice each). The gray shading represents the physical dimensions of each subnuclei in the rostral–caudal axis (positive values denote rostral of obex, negative values denote caudal of obex; Paxinos and Franklin, 2012).

High-magnification images were made of medulla following staining of intrinsic neuron soma with the fluorescent Nissl stain neurotrace (Figs. 13, 14). Reporter expression was observed throughout the entire of the nTS of the Pirt-tdT (Fig. 13*A*), with very strong labeling of fibers within the commissural subnucleus (SolC), gelatinous subnucleus (SolG), dorsal lateral subnucleus (SolDL), medial subnucleus (SolM), and the tractus solitarius, and strong labeling of fibers in the intermediate subnucleus (SolIM), ventral subnucleus (SolV), and ventrolateral subnucleus (SolVL). There were also tdTomato^+^ fibers crossing into the area postrema and the DMX. In addition, numerous intrinsic neurons expressing tdTomato were found throughout the area postrema and lining the central canal. A few tdTomato-expressing neurons were also found in the nTS and DMX. Similar to the Pirt-tdTomato, reporter expression was observed throughout the entire 5-HT3-tdT nTS (Fig. 13*B*), with very strong labeling of fibers in the SolC, SolG, and the tractus solitarius, and strong labeling of fibers in SolM, SolIM, SolDL, SolV, and SolVL. Virtually no nTS neurons expressed tdTomato, but there were some reporter-expressing fibers crossing into the area postrema and the DMX. tdTomato expression in the TRPV1-tdT was very strong in SolC, SolG, tractus solitarius, SolDL, and, to a lesser degree, in SolM and the area postrema (Fig. 13*C*). Although limited, we also observed reporter expression in fibers within SolIM, SolV, SolVL, and DMX. There was sporadic tdTomato expression in intrinsic neurons throughout the nTS and DMX, but not in the area postrema. Reporter expression in the dorsal medulla of Tac1-tdT mice was widespread in both fibers and intrinsic neurons (Fig. 13*D*). Numerous intrinsic neurons within SolM, SolDL, SolIM, SolV, SolVL, and the hypoglossal nucleus expressed tdTomato, with a small subset of intrinsic neurons in the area postrema also expressing the reporter. SolC, SolG, central subnucleus, (SolCe) and the DMX had very few tdTomato-expressing neurons. In general, the areas with more tdTomato-expressing neurons had more tdTomato-expressing fibers, although there was also substantial innervation of the DMX with tdTomato-expressing fibers. Despite their low abundance compared with the rest of the nTS, we nevertheless observed tdTomato-expressing fibers within SolC, SolG, and the area postrema.

**Figure 15.**
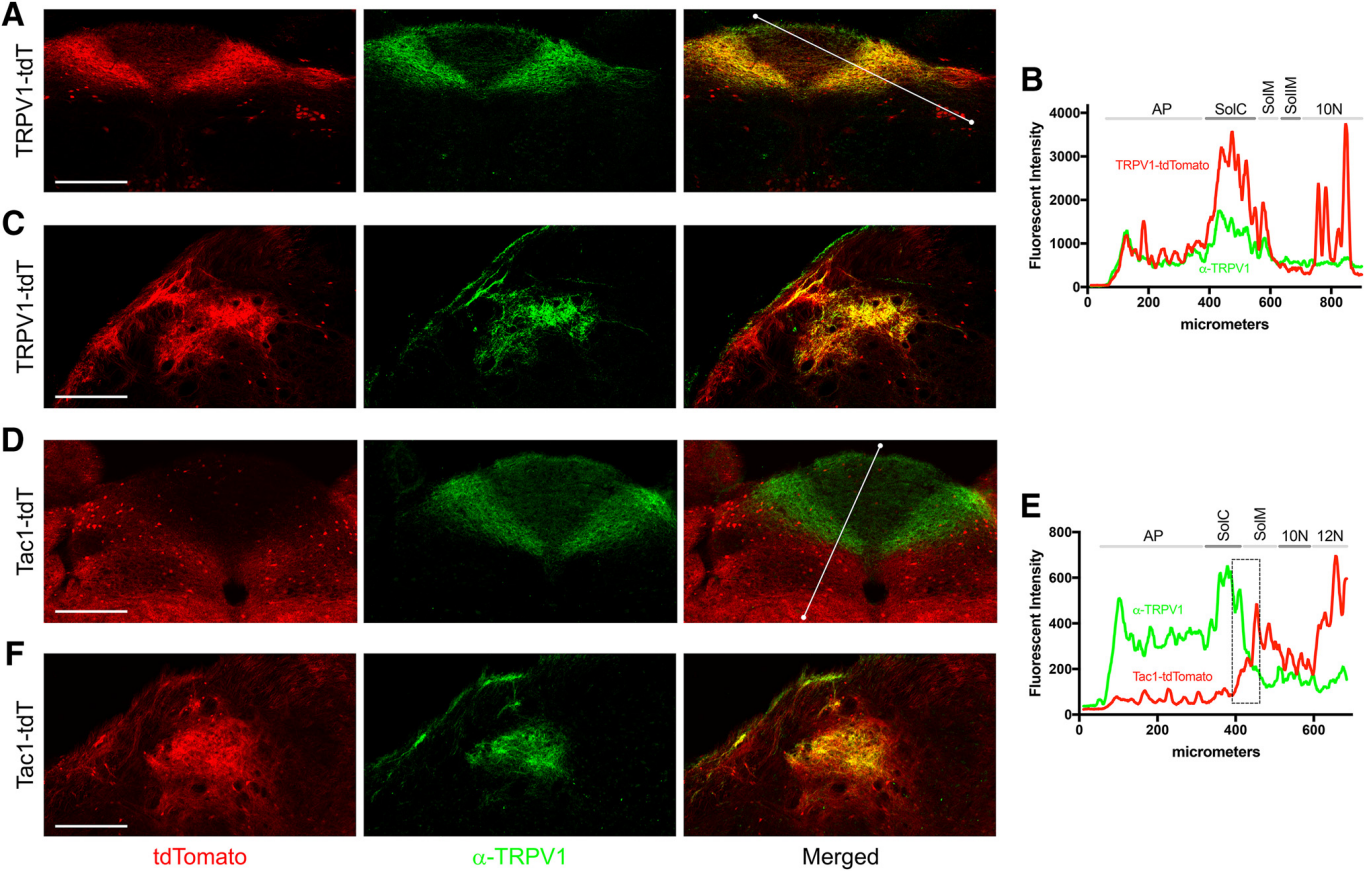
Overlap of native tdTomato expression and α-TRPV1 immunoreactivity in the medulla of TRPV1-tdT and Tac1-tdT. ***A*–*C***, TRPV1-tdT at −300 μm (relative to obex). ***D*–*F***, Tac1-tdT at −240 μm. ***A***, ***C***, ***D***, ***F***, tdTomato expression (red) is compared with α-TRPV1 immunoreactivity (green) in the nTS (***A*–*D***) and the Pa5 (***C*–*F***), with merged image on the right. Scale bar, 250 μm. *B*, Quantification of tdTomato (red) and α-TRPV1 immunoreactivity (green) intensities along the line drawn in the merged image in ***A*** (TRPV1-tdT nTS), including area postrema (AP), dorsal motor nucleus of the vagus (10N), SolC (C), SolIM (IM), and SolM (M). ***E***, Quantification of tdTomato (red) and α-TRPV1 immunoreactivity (green) intensities along the line drawn in the merged image in ***D*** (Tac1-tdT nTS), including AP, 10N, 12N, C, and M. Boxed area denotes overlap of tdTomato and α-TRPV1 immunoreactivity intensities along the border between SolC and SolM. Data are representative of *n* = 3 animals for each strain.

Given that transient embryological Cre expression causes reporter expression regardless of gene expression in the adult, we used immunofluorescence to determine whether the reporter expression within the nTS, DMX, hypoglossal nucleus, and Pa5 of TRPV1-tdT, as seen in Figures 10 and 13*C*, was indicative of adult expression of TRPV1. We found significant overlap between tdTomato-expressing fibers in the medial nTS subnuclei of TRPV1-tdT and α-TRPV1 immunoreactivity (Fig. 15*A*,*B*). Similarly, TRPV1-tdT^+^ fibers in the Pa5 also had α-TRPV1 immunoreactivity (Fig. 15*C*). Importantly, none of the tdTomato-expressing intrinsic neurons had α-TRPV1 immunoreactivity (Fig. 15*A*,*C*), indicating that these neurons did not express TRPV1 in the adult mouse. We also noted that there was a subset of axons within the tractus solitarius of TRPV1-tdT that did not have α-TRPV1 immunoreactivity, but this group did not appear to innervate a particular nTS subnucleus.

Our vagal ganglia data indicated that some of Tac1-tdT^+^ vagal neurons (particularly in the jugular ganglia) also had α-TRPV1 immunoreactivity (Fig. 1). However, our Tac1-tdT data indicated limited reporter expression in medial nTS subnuclei (SolC and SolG) robustly innervated by tdTomato-expressing fibers in the TRPV1-tdT (Fig. 13). Nevertheless, we found that there was a narrow band at the border of SolC and SolM of tdTomato-expressing fibers in the Tac1-tdT that had substantial α-TRPV1 immunoreactivity (Fig. 15*D*,*E*, dashed box). We also found substantial overlap between reporter expression in the Tac1-tdT within the Pa5 and α-TRPV1 immunoreactivity (Fig. 15*F*).

### AAV-mediated reporter expression in TRPV1-Cre and Tac1-Cre mice

**Figure 16.**
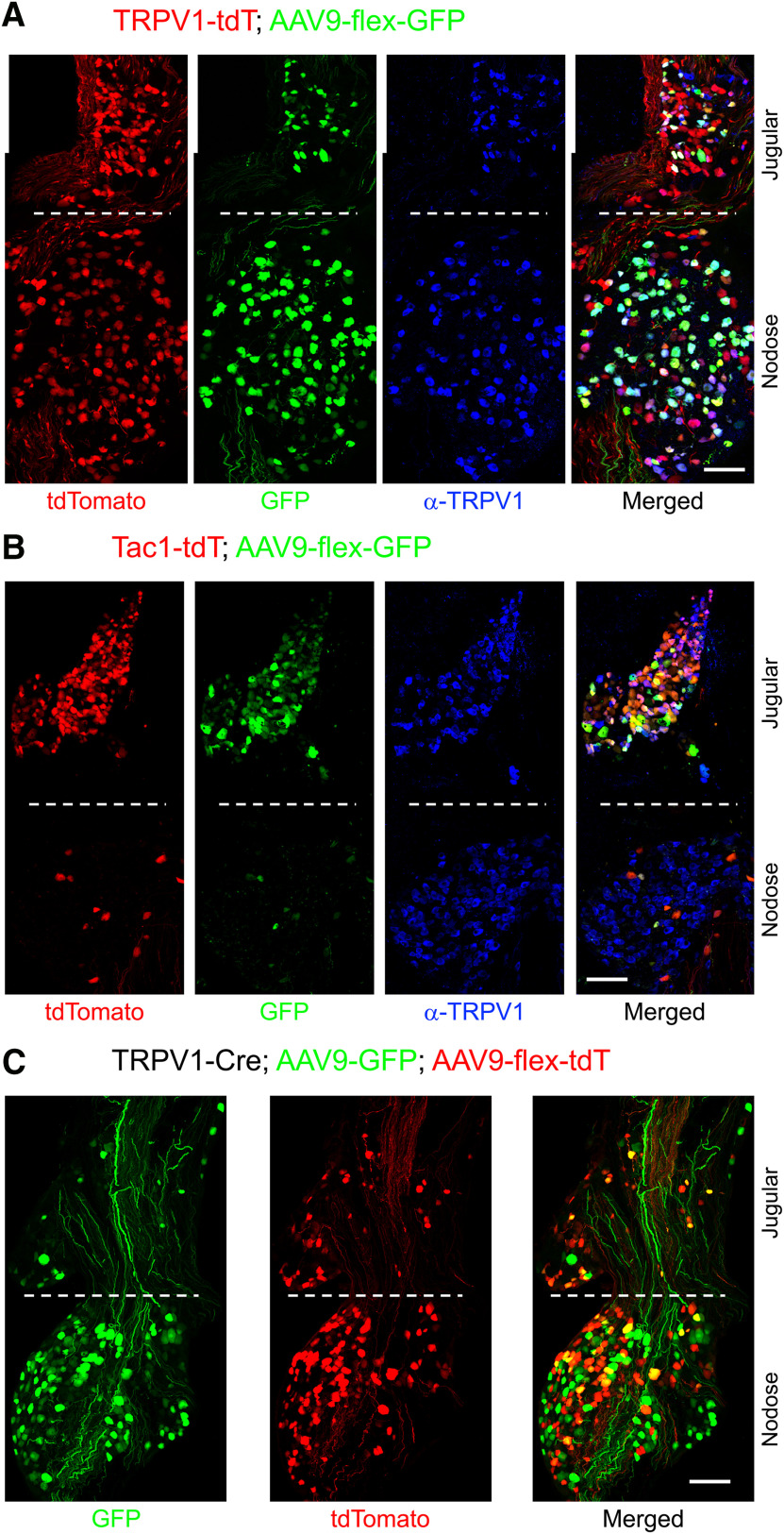
Intraganglionic injection of AAV vectors evokes robust reporter expression in vagal ganglia. ***A***, Injection of AAV9-flex-GFP into TRPV1-tdT. tdTomato (red), and GFP (green) expression is compared with α-TRPV1 immunoreactivity (blue). ***B***, Injection of AAV9-flex-GFP into Tac1-tdT. tdTomato (red) and GFP (green) expression is compared with α-TRPV1 immunoreactivity (blue). ***C***, Coinjection of AAV9-flex-tdT and AAV9-GFP into TRPV1-Cre. tdTomato (red) and GFP (green) expression is compared. Scale bar, 100 μm.

In order to specifically visualize the central terminals of vagal afferent subsets, we used an AAV vector approach. We first unilaterally injected a Cre-sensitive AAV reporter (AAV9-flex-GFP) into the left vagal ganglia of TRPV1-tdT and Tac1-tdT mice, which was expected to induce GFP expression in neurons that currently expressed Cre. We observed GFP labeling in vagal neurons from four of six TRPV1-tdT mice injected with virus (Fig. 16*A*): 1093 of 1627 tdTomato^+^ neurons (67%) expressed GFP, and only 9 of 1111 GFP^+^ neurons (<1%) lacked either tdTomato expression or α-TRPV1 immunoreactivity. We observed GFP labeling in vagal neurons from five of eight Tac1-tdT mice injected with virus (Fig. 16*B*): 448 of 746 tdTomato^+^ neurons (60%) expressed GFP, and only 11 of 459 GFP^+^ neurons (2.4%) lacked tdTomato expression (Fig. 16*B*). In another study, we unilaterally injected a mixture of constitutively active AAV9-GFP and the Cre-sensitive AAV9-flex-tdT into the left vagal ganglia of TRPV1-Cre mice in order to compare the central innervation by TRPV1^+^ and TRPV1^−^ vagal afferents. We observed reporter expression in vagal neurons from two of two TRPV1-Cre mice injected with both viruses. Specifically, we observed GFP labeling in 659 neurons, 463 of which (70%) also expressed tdTomato (Fig. 16*C*). Only 11 of 474 tdTomato^+^ neurons (2.3%) lacked GFP expression. A subset of GFP^+^ axons lacking tdTomato in the vagal ganglia was visibly thicker than the GFP^+^/tdTomato^+^ axons. In all AAV9 studies, no AAV-mediated reporter expression was noted in any cell types other than afferent neurons, and none was noted in the right vagal ganglia (no injection).

**Figure 17.**
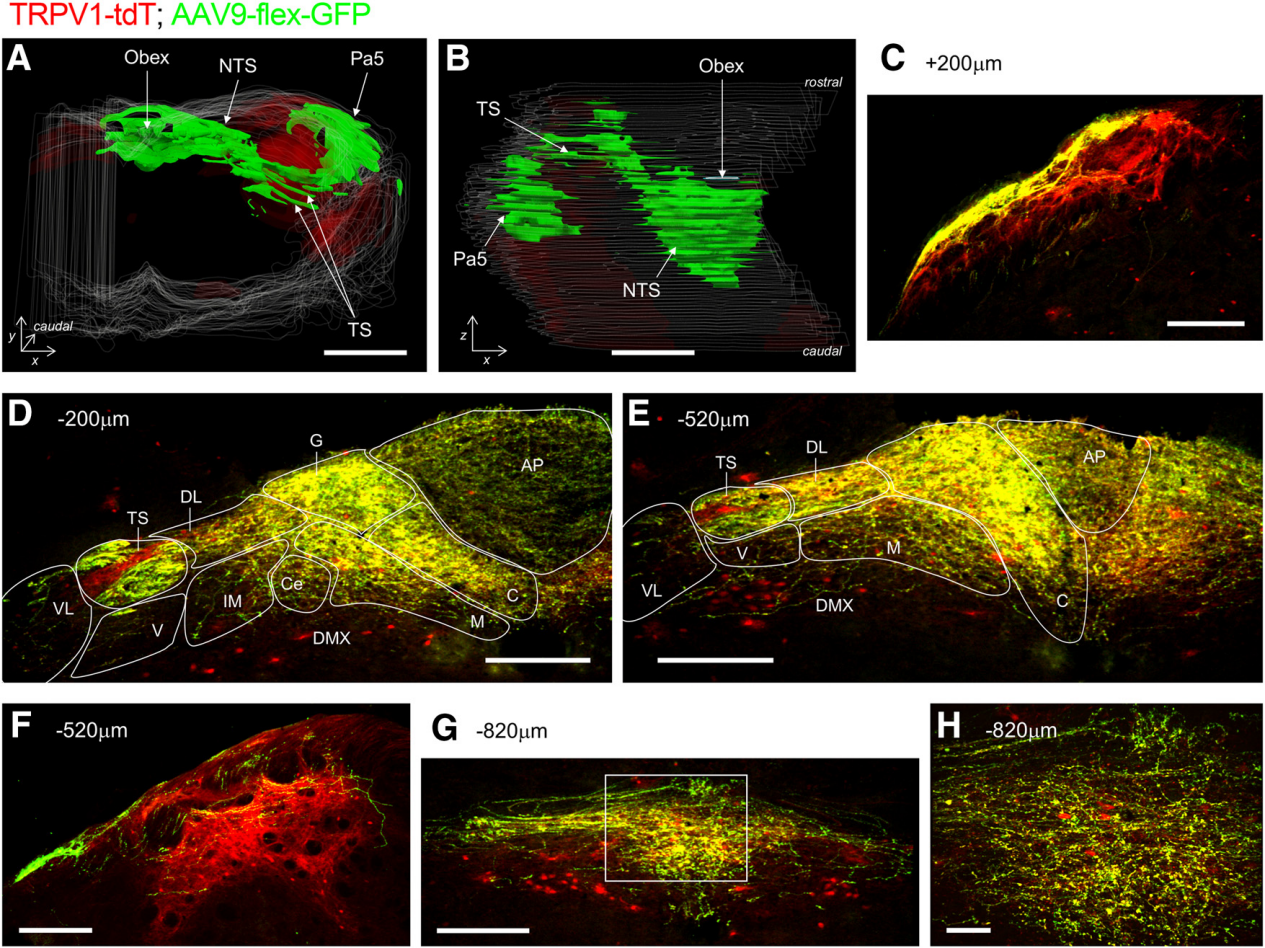
Brainstem terminations of vagal TRPV1-expressing afferents labeled by unilateral intraganglionic injection of AAV9-flex-GFP into TRPV1-tdT. AAV-mediated GFP expression (green, enhanced by α-GFP immunoreactivity) is compared with ROSA26-mediated tdTomato expression (red, native) in coronal sections of the medulla. ***A***, ***B***, 3D reconstruction of medulla along entire rostral–caudal axis. ***A***, Rostral aspect. ***B***, Dorsal aspect. ***C*–*H***, Coronal sections of medulla from rostral to caudal, with labeling for the position relative to obex. ***C***, Pa5 at +300 μm (relative to obex). ***D***, ***E***, nTS at −200 μm (***D***) and −520 μm (***E***). The following structures are identified: area postrema (AP), DMX, SolC (C), SolCe (Ce), SolDL (DL), SolG (G), SolIM (IM), SolM (M), SolV (V), SolVL (VL), and TS. ***F***, Pa5 at −520 μm. ***G***, ***H***, nTS at −820 μm. ***H***, High-magnification image of SolC area identified by white box in ***G***. Scale bars: ***A***, ***B***, 1 mm; ***C–G***, 200 μm; ***H***, 40 μm.

Serial sections of the medulla from AAV9-flex-GFP-treated TRPV1-tdT mice indicated that vagal TRPV1-expressing (GFP^+^) central terminations bilaterally innervated the nTS, area postrema, and, to a limited extent, the DMX (Fig. 17), and unilaterally innervated the tractus solitarius and the Pa5. With the exception of an occasional bundle of axons within the ipsilateral tractus solitarius, there were no areas within the ipsilateral nTS that had an abundance of tdTomato-expressing GFP-negative fibers. This suggests that the subset of TRPV1-tdT^+^ vagal neurons that lack adult expression of TRPV1 do not preferentially innervate a particular location within the nTS. Consistent with our previous data from TRPV1-tdT, GFP^+^ fibers most densely innervated SolC, SolG, and SolDL (from +40 to −1120 μm, relative to obex), with fewer GFP^+^ fibers in SolM and the area postrema, and only sporadic innervation of SolIM, SolV, SolVL, and the DMX (Fig. 17*D*,*E*,*G*,*H*). Contralateral innervation by GFP^+^ fibers was robust but clearly less than ipsilateral innervation. There was significant innervation by GFP^+^ fibers within the ipsilateral Pa5, although these fibers were a minor population compared with other tdTomato-expressing nerves (Fig. 17*C*,*F*).

**Figure 18.**
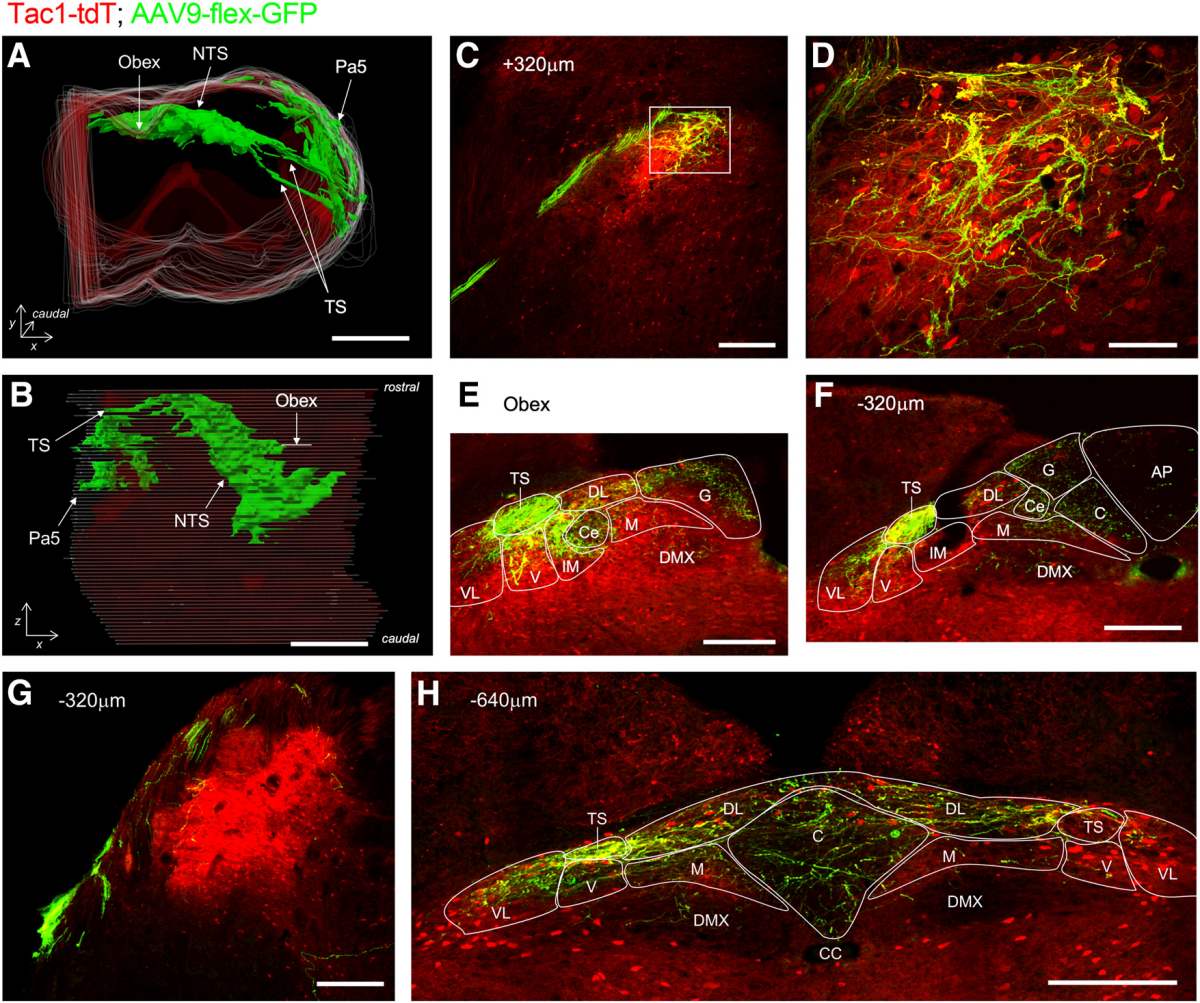
Brainstem terminations of vagal Tac1-expressing afferents labeled by unilateral intraganglionic injection of AAV9-flex-GFP into Tac1-tdT. AAV-mediated GFP expression (green, enhanced by α-GFP immunoreactivity) is compared with ROSA26-mediated tdTomato expression (red, native) in coronal sections of the medulla. ***A***, ***B***, 3D reconstruction of medulla along entire rostral–caudal axis. ***A***, Rostral aspect. ***B***, Dorsal aspect. ***C–H***, Coronal sections of medulla from rostral to caudal, with labeling for the position relative to obex. ***C***, nTS at +320 μm (relative to obex). ***D***, High-magnification image of area identified by white box in ***C***. ***E***, ***F***, nTS at obex (***E***) and −320 μm (***F***). ***G***, Pa5 at −320 μm. ***H***, nTS at −640 μm. The following structures are identified: area postrema (AP), central canal (CC), DMX, Pa5, SolC (C), SolCe (Ce), SolDL (DL), SolG (G), SolIM (IM), SolM (M), SolV (V), SolVL (VL), and TS. Scale bars: ***A***, ***B***, 1 mm; ***C***, ***E***, ***G***, 200 μm; ***D***, 50 μm.

AAV9-flex-GFP treatment of Tac1-tdT mice induced GFP expression in central terminations innervating the nTS, Pa5, and, to a limited extent, the DMX and area postrema (Fig. 18). Again, the GFP-expressing fibers were observed bilaterally in the nTS, area postrema, and DMX but were only noted unilaterally in the tractus solitarius and the Pa5. GFP^+^ fibers were observed in the rostral nTS (+520 μm, relative to obex) through to the caudal nTS (−900 μm), in many cases directly innervating Tac1-tdT^+^ intrinsic neurons (Fig. 18*C*,*D*). The tdTomato expression in the numerous reporter-expressing intrinsic neurons clearly exceeded the signal from the tdTomato-expressing vagal afferents. Consistent with AAV being unable to infect nerves transsynaptically, there was no GFP expression in tdTomato-expressing intrinsic cells in the medulla. Nevertheless, the AAV-mediated GFP expression indicated that vagal Tac1-expressing afferents innervated ipsilateral SolC, SolG, SolDL, SolCe, SolM, SolIM, SolV, and SolVL (Fig. 18*D–F*,*H*). Innervation of the contralateral nTS with GFP^+^ fibers was observed only in areas caudal to obex (−40 to −840 μm, relative to obex), and this was mostly in the more dorsal and medial subnuclei (SolG, SolC, and SolDL; Fig. 18*A*,*B*,*H*).

**Figure 19.**
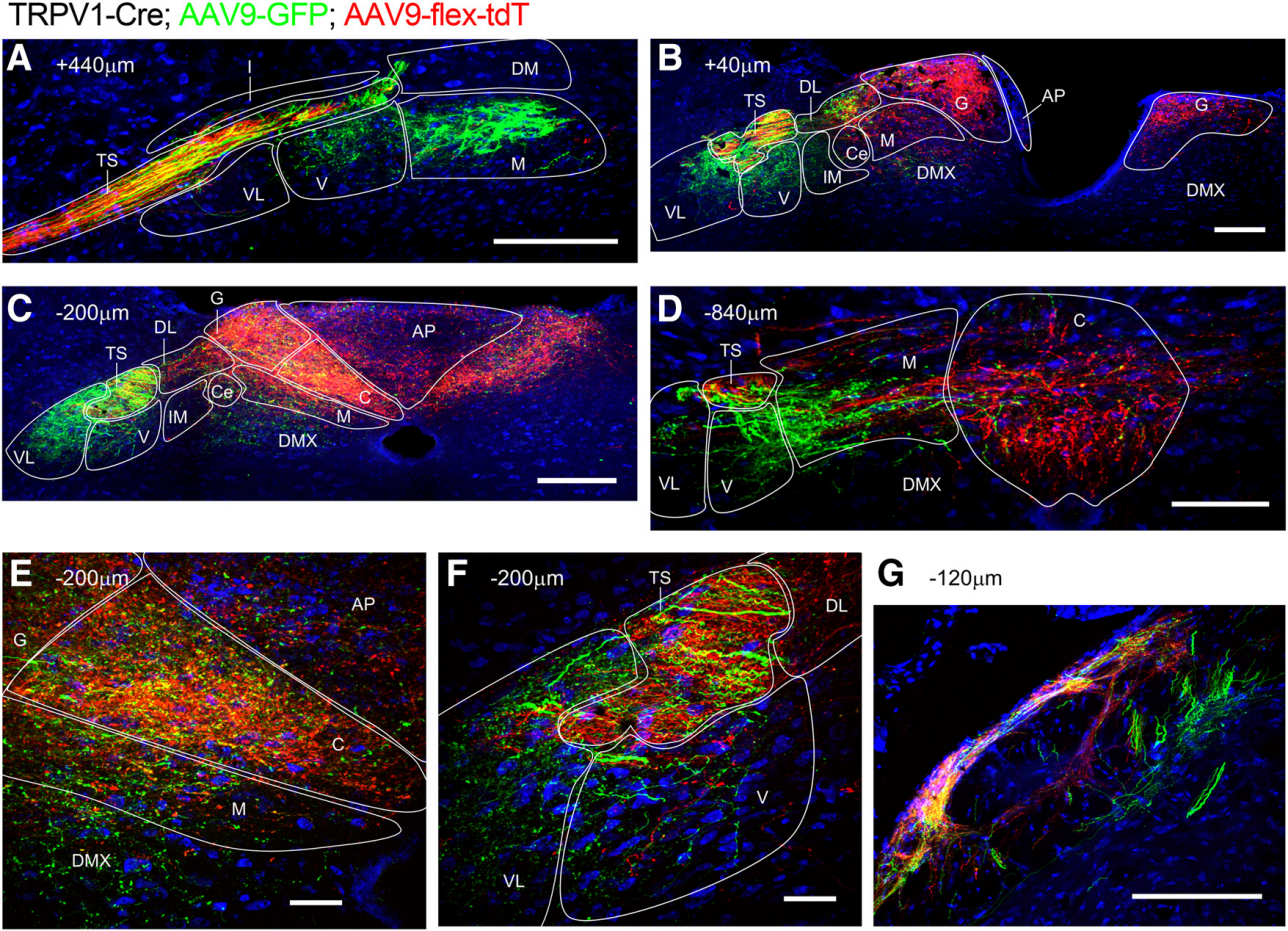
Brainstem terminations of vagal TRPV1^+^ and TRPV1^−^ afferents labeled by unilateral intraganglionic injection of AAV9-flex-tdT and AAV9-GFP into TRPV1-Cre. Cre-sensitive AAV-mediated tdTomato expression (red; enhanced by α-DsRed immunoreactivity) is compared with constitutively active AAV-mediated GFP expression (green, enhanced by α-GFP immunoreactivity) and neurotrace (blue) in coronal sections of the medulla (labeling for the position relative to obex). ***A*–*D***, nTS from rostral to caudal. ***E***, High-magnification image of SolC, SolM, and AP from ***C***. ***F***, High-magnification image of SolV, SolVL, and TS from ***C***. ***G***, Pa5 at −120 μm. The following structures are identified: area postrema (AP), DMX, SolC (C), SolCe (Ce), SolDL (DL), SolG (G), SolIM (IM), SolM (M), SolV (V), SolVL (VL), and TS. Scale bars: ***A***, ***B***, ***C***, ***G***, 200 μm; ***D***, 100 μm; ***E***, ***F***, 40 μm.

Unilateral treatment of TRPV1-Cre mice with a combination of constitutively active AAV9-GFP and the Cre-sensitive AAV9-flex-tdT demonstrated the differential central terminations of vagal TRPV1-expressing afferents (expressing tdTomato) and afferents lacking TRPV1 (expressing only GFP). As before, tdTomato^+^ TRPV1-expressing vagal afferents were mostly found terminating bilaterally in the medial and superficial nTS regions (SolG, SolC, and SolDL) at the level of obex and more caudally (Fig. 19*A*–*F*). Whereas, the majority of fibers expressing GFP alone (TRPV1^−^ afferents) terminated ipsilaterally in more lateral and ventral areas such as SolVL, SolV, and SolM (and to some extent the DMX) throughout the entire rostral–caudal axis (+480 to −960 μm; Fig. 19*A*–*F*). Nevertheless, a small number of terminations expressing GFP alone were noted bilaterally in the caudal SolC. Along with the expected ipsilateral tdTomato^+^ fibers of TRPV1-expressing afferents within the Pa5, we also noted Pa5 terminations that only expressed GFP (Fig. 19*G*), suggesting that TRPV1^−^ vagal afferents also innervate this medulla region.

**Figure 20.**
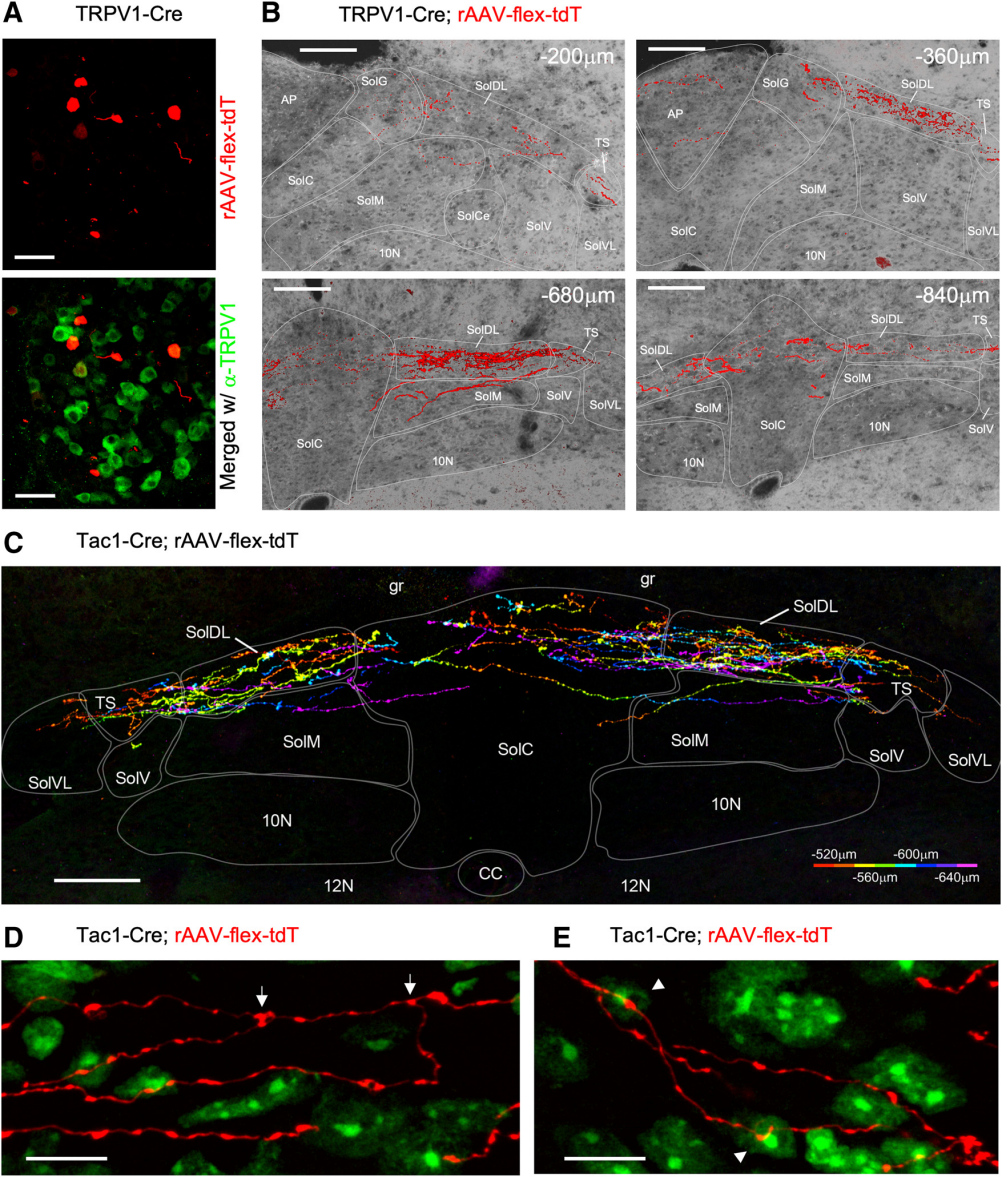
nTS terminations of lung-specific TRPV1^+^ and Tac1^+^ afferents labeled with rAAV-flex-tdT. tdTomato expression enhanced by enhanced by α-DsRed immunoreactivity. ***A***, ***B***, Instillation of Cre-sensitive AAV into lungs of TRPV1-Cre. ***A***, AAV-mediated tdTomato expression (red) identifying lung-specific TRPV1^+^ afferents in vagal ganglia (top) and merged with α-TRPV1 immunoreactivity (green; bottom). *B*, AAV-mediated tdTomato expression (red) in coronal sections of the medulla (labeling for the position relative to obex), counterstained with native autofluorescence (gray). ***C*–*E***, instillation of Cre-sensitive AAV into lungs of Tac1-Cre. ***C***, Composite image of serial coronal sections of the nTS (from −520 to −640 μm relative to obex), with tdTomato expression in lung-specific Tac1^+^ afferents labeled in pseudorainbow encoded by rostral–caudal position. ***D***, ***E***, High-magnification images of lung-specific Tac1^+^ afferents (red) in SolC at −520 μm, counterstained by neurotrace (green). ***D***, Branching of afferent denoted by arrows. ***E***, A single afferent makes putative synapses with two distinct intrinsic neurons, denoted by arrowheads. Contact SolC, SolM, and AP from ***C***. ***F***, High-magnification image of SolV, SolVL, and TS from ***C***. The following structures are identified: area postrema (AP), dorsal motor nucleus of the vagus (10N), gracile fasciculus (g), 12N, SolC (C), SolCe (Ce), SolDL (DL), SolG (G), SolM (M), SolV (V), SolVL (VL), and TS. Scale bars: ***A***, 50 μm; ***B***, ***C***, 100 μm; ***D***, ***E***, 10 μm.

In order to trace subset-specific afferents from the lower airways, we instilled a Cre-sensitive retrograde AAV reporter (rAAV-flex-tdT) into the lungs of TRPV1-Cre and Tac1-Cre mice. The rAAV-flex-tdT induced tdTomato in a limited number of nodose and jugular neurons bilaterally in the vagal ganglia of TRPV1-Cre mice (five of six animals had tdTomato^+^ vagal neurons; range, 6–31 neurons per mouse) and Tac1-Cre mice (three of three animals had tdTomato^+^ vagal neurons; range, 2–12 neurons per mouse; Fig. 20*A*). In the medulla, individual axons expressing tdTomato were observed within the tractus solitarius as the pathway invades the ventral aspect of the lateral medulla wall and proceeds medially toward the nTS. This was detected between +120 and −120 μm (relative to obex) for TRPV1-Cre, and between +240 and −200 μm for Tac1-Cre mice (data not shown). In the TRPV1-Cre mice, rAAV-flex-tdT induced tdTomato expression in fibers within SolDL, SolG, and SolC between −200 and −960 μm (relative to obex), with the majority of terminations occurring from −400 to −840 μm (Fig. 20*B*). In addition, rAAV-flex-tdT induced limited tdTomato expression in terminations in SolM and the area postrema, but not in SolCe, SolV, SolVL, or the DMX (Fig. 20*B*). The reporter-labeled fibers in the Tac1-Cre mice were in general brighter than those in the TRPV1-Cre mice. In the Tac1-Cre mice, rAAV-flex-tdT induced tdTomato expression in terminations within SolDL, SolG, and SolC between −240 and −800 μm, with the majority of terminations occurring from −440 to −680 μm (Fig. 20*C*). rAAV-flex-tdT induced reporter expression in a limited number of terminations within the Tac1-Cre SolM and SolVL, but there was no labeling of terminations in either the area postrema or the DMX (Fig. 20*C*). The reporter-labeled fibers were sparse enough to investigate their relationship with intrinsic neurons (labeled with neurotrace) within the nTS. We noted numerous branching of lung-specific Tac1^+^ terminations (Fig. 20*D*). Furthermore, we observed individual lung-specific Tac1^+^ fibers with varicosities in close proximity to multiple neurons (Fig. 20*E*). No structures within the Pa5, Sp5, or hypoglossal nucleus were labeled by tdTomato in either TRPV1-Cre or Tac1-cre mice (data not shown).

## Discussion

Vagal sensory nerves are heterogeneous with respect to gene expression, embryological source, stimuli sensitivity, size, and their extent of myelination (Ricco et al., 1996; Zhuo et al., 1997; Yu et al., 2005; Nassenstein et al., 2010; Wang et al., 2017; Kupari et al., 2019). Furthermore, activation of unique subsets of vagal afferents evokes specific reflex behaviors and sensations (Thoren, 1979; Mazzone and Canning, 2002; Carr and Undem, 2003; Mazzone et al., 2005; Chou et al., 2008, 2018; Chang et al., 2015; Hooper et al., 2019). Here, we have used a series of genetic reporters to identify differences between the central terminations of specific vagal afferent subsets.

Consistent with data from DRG afferents (Patel et al., 2011), Pirt is expressed on almost all nodose and jugular afferents, with a nodose to jugular frequency ratio of 2.3:1, consistent with previous counts of afferent neurons within the vagal ganglia (Mazzone et al., 2020). Thus, Pirt-tdT is a useful marker of all vagal afferents. The 5-HT3-tdT mouse labeled virtually all nodose neurons, but very few jugular neurons were labeled. 5-HT3-selective agonists have been shown to activate nodose C-fibers innervating the airways, but these ligands fail to activate jugular C-fibers (Chuaychoo et al., 2005). There is also evidence that 5-HT3-selective agonists can activate some airway A-fibers (fibers that lack TRPV1 expression; Ho et al., 2001). These functional studies are supported by RNA transcriptomics of vagal neurons (Wang et al., 2017; Kupari et al., 2019) and airway-specific vagal neurons (Mazzone et al., 2020) that indicate that high levels of 5-HT3 transcripts are found in nodose neuronal clusters (both TRPV1^+^ and TRPV1^−^ clusters), but not in jugular neuronal clusters.

Consistent with previous studies of functional TRPV1 expression in vagal neurons (Undem et al., 2004; Nassenstein et al., 2010; Hooper et al., 2016), a substantial subset of nodose and jugular neurons were TRPV1-tdT^+^. Importantly, only 71% of nodose TRPV1-tdT^+^ neurons and 50% of jugular TRPV1-tdT^+^ neurons also had α-TRPV1 immunoreactivity. Furthermore, intraganglionic injection of TRPV1-tdT mice with cre-sensitive AAV9-flex-GFP resulted in GFP expression in 67% of tdT^+^ neurons (although this may be impacted by a <100% transfection efficiency). These data are largely consistent with the reported 61% of dissociated vagal TRPV1-tdT^+^ neurons that were sensitive to the TRPV1-selective agonist capsaicin (Stanford et al., 2019). Thus, like DRG afferents (Cavanaugh et al., 2011; Patil et al., 2018), there appears to be a population of vagal afferents in mice that only transiently express TRPV1 during development. Little is known of their function. Nevertheless, our data show that the TRPV1-Cre is a useful marker for nociceptors from both the nodose (TRKB-expressing) and jugular (TRKA-expressing) ganglia. Last, the Tac1-tdT labeled a heterogeneous population of vagal neurons. The majority of Tac1-tdT^+^ neurons were found in the jugular ganglia, and these often coexpressed TRKA and TRPV1. Whereas the Tac1-tdT^+^ neurons in the nodose ganglia were limited in number and very rarely coexpressed TRPV1 or TRKB. As such, it is likely that Tac1-tdT labels a subset of jugular TRPV1^+^ neurons and a subset of nodose TRPV1^−^ neurons. Transcriptomic analysis shows high levels of Tac1 in jugular TRPV1-expressing neurons compared with nodose TRPV1-expressing neurons (Nassenstein et al., 2010; Wang et al., 2017). Interestingly, cluster analysis of vagal neurons by Kupari et al. (2019) identified a subset of nodose neurons (termed NG5) that had substantial Tac1 expression, but had very low expression for TRKA, TRKB, TRPV1, TRPA1, and TRPM8. Intraganglionic injection of AAV-flex-GFP induced GFP expression in 60% of tdTomato-expressing neurons in the Tac1-tdT. It is presently unclear why this number is <100%, but may either indicate transient expression of Tac1 during development or suggest that AAV transfection was not completely efficient.

It is important to note that the nodose and jugular ganglion are fused in mice, unlike in guinea pigs and larger mammals. As such, the designation of nodose versus jugular was determined for each neuron subjectively (although blinded to the marker being evaluated) based on the idiosyncratic gross anatomy of each ganglion. This is a limitation of the study, as previous studies of selective labeling of Wnt1-expressing neural-crest/jugular neurons showed that jugular and nodose neurons are not perfectly delineated in the vagal ganglia (Nassenstein et al., 2010; Surdenikova et al., 2012). Nevertheless, the current designations are likely reasonably suitable for our analysis as the selective expression of TRKA and TRKB in the “nodose” and “jugular” groups are consistent with the distinct expression of these markers in neural crest and placodal neurons (Nassenstein et al., 2010; Lieu et al., 2011; Wang et al., 2017; Kupari et al., 2019).

Our medulla data indicate that there is preferential innervation of specific nTS areas by specific afferent subsets. The all-afferent marker, Pirt-tdT, labeled central terminals along the entire rostral–caudal axis of the nTS, and, although the most densely innervated areas were the medial subnuclei such as SolC and SolG, ventral and lateral subnuclei such as SolV and SolVL were also strongly labeled. Similar nTS labeling was noted for 5-HT3-tdT, the marker for nodose afferents. Whereas vagal TRPV1-expressing afferents terminated overwhelmingly in the medial/dorsal subnuclei (SolC, SolG, and SolDL), and this labeling was noted almost exclusively at obex and more caudal regions. Other caudal subnuclei, including SolM, SolV, and SolVL, were nonetheless labeled sparsely by vagal TRPV1-expressing afferents. This data are consistent with the pattern of vagal afferent terminations within the nTS produced using AAV-mediated reporter expression driven by the Npy2r gene (Chang et al., 2015), which is strongly correlated with TRPV1 expression. Comparison of tdTomato and GFP expression in the nTS of TRPV1-tdT with intraganglionic injection of AAV9-flex-GFP suggests that central terminals expressing tdTomato only (either vagal afferents transiently expressing TRPV1 in development or nonvagal TRPV1-expressing afferents) innervate the same subnuclei as vagal TRPV1-expressing afferents.

Using intraganglionic injection of TRPV1-Cre mice with both constitutively active AAV9-GFP and Cre-sensitive AAV9-flex-tdT, we have attempted to identify the terminations of afferents that lack TRPV1. The majority of these apparently TRPV1^−^ afferents terminated in lateral and ventral regions of the nTS (e.g., SolM, SolVL, and SolV), and these were present throughout the entire rostral–caudal axis. We also noted some TRPV1^−^ terminations within the caudal SolC. Nevertheless, the determination of “TRPV1^−^” in these studies was based on GFP expression without tdTomato expression and thus may simply be due to inefficiency in the AAV9-flex-tdT. We believe this is unlikely to be a major concern given the efficiency of AAV9-mediated reporter expression noted in the vagal ganglia, and due to the distinct termination patterns of AAV9-GFP and AAV9-flex-tdT.

Interestingly, lung-specific TRPV1-expressing afferents (identified using rAAV2 vector) only terminated in SolC, SolDL, and SolG (and to a minor extent SolM), and these were only found caudal to obex (−200 to −960 μm). This suggests a degree of organotopic organization, but this hypothesis requires direct comparison with TRPV1-expressing afferents from other organs. Lung-specific TRPV1-expressing afferents did not appear to terminate in areas outside the nTS, such as the Pa5 (see below for further discussion). Compared with the efficiency of the intraganglionic injection of AAV9 in inducing reporter expression, the number of vagal neurons with reporter expression driven by the rAAV2 vector instilled into the airways was limited, and likely represents a minority of the total number of vagal afferents innervating the lower airways. This inefficiency may be due to the limited number of vector particles internalized by the afferent terminals beneath the epithelial lining of the airways. It is possible that some upper airway vagal afferents may also have been transduced by the rAAV2 due to expulsion of the virus from the lower airways, but we noted no trigeminal fiber transduction (i.e., nasal). Transduction of esophageal afferents was highly unlikely given the impermeability of the esophageal lining.

Overall, the rostral versus caudal and lateral versus medial distinctions between TRPV1^−^ afferent terminations and TRPV1^+^ afferent terminations observed in this present mouse study are consistent with electrophysiological recordings of airway-associated afferents within the nTS of rats, cats, and rabbits: slowly adapting receptors (SARs), a class of lung TRPV1^−^ stretch-sensitive A-fibers, terminate in SolVL and SolM, particularly rostral of obex (Donoghue et al., 1982; Davies and Kubin, 1986; Davies et al., 1987; Bonham and McCrimmon, 1990); rapidly adapting receptors (RARs), another stretch-sensitive A-fiber subset that lacks TRPV1 expression, terminate in SolVL and SolM (although these are rare rostral of obex) and also in the ventral and lateral parts of SolC (Davies and Kubin, 1986; Lipski et al., 1991); finally, bronchopulmonary C-fibers (which typically express TRPV1) largely terminate in SolC and SolG (Kubin et al., 1991). As such, it appears there are significant similarities between the terminations identified using genetic tools in the mouse and the terminations identified electrophysiologically in larger mammals whose respiratory reflexes have been more extensively studied.

The expression of Tac1 in many intrinsic neurons within the medulla precluded conclusions regarding afferent terminations in the Tac1-tdT medulla but, using AAV9-mediated reporter expression, we found vagal Tac1-expressing terminals in subnuclei throughout the entire rostral–caudal axis of the nTS. Nevertheless, these terminals were relatively sparse. In the vagal system, our data suggest that Tac1 is a marker of both jugular TRPV1^+^ neurons and a subset of nodose afferents lacking TRPV1. Based on the coincidence of TRPV1 and Tac1 vagal afferents within caudal subnuclei such as SolC, SolDL, and SolG, it is likely (but not proven) that the Tac1-expressing terminations in these areas also express TRPV1. The lack of TRPV1-expressing fibers in rostral, ventral, or lateral areas suggests that the Tac1-expressing fibers in these areas are likely to be TRPV1^−^. Although most nodose Tac1^+^ neurons lacked TRPV1, many jugular Tac1^+^ neurons also lacked TRPV1, so it is not possible at this time to determine the identity of the rostral terminations. Interestingly, lung-specific Tac1^+^ afferents almost exclusively terminated within the caudal SolC, SolDL, and SolG regions (similar to lung-specific TRPV1^+^ terminations). Thus, our data suggest that the vast majority of lung-specific Tac1^+^ innervation is derived from jugular TRPV1^+^ afferents, consistent with biochemical studies of lung-specific vagal afferents (Ricco et al., 1996; Undem et al., 2004; Nassenstein et al., 2010) and the impact of SolC microinjections of tachykinin ligands on respiratory function (Mazzone and Geraghty, 1999, 2000). It is not currently known which organs have nodose Tac1^+^/TRPV1^−^ afferents that terminate in the rostral nTS.

Unilateral intraganglionic injection of AAV reporters induced bilateral labeling of afferent terminations within the nTS, indicating collateral arborizations. Interestingly, the majority of the contralateral terminations were TRPV1^+^, which terminated in dorsal/medial subnuclei. There were only a few contralateral TRPV1^−^ terminations, and these did not project to rostral, ventral, or lateral areas. These data are consistent with electrophysiological recordings that suggest that while bronchopulmonary C-fibers and RARs innervate the nTS bilaterally, SARs only innervate the ipsilateral nTS (Donoghue et al., 1982; Davies and Kubin, 1986; Davies et al., 1987; Kubin et al., 1991).

Recently, the Pa5 has been identified as potentially receiving direct input from vagal afferents—specifically jugular neurons (McGovern et al., 2015b). The Pa5 is thought to receive input from trigeminal, vagal, glossopharyngeal, and DRG afferents, and projects to multiple nuclei involved in autonomic and nociceptive processing in the medulla and pons and may also project to the somatosensory thalamus (McGovern et al., 2015b; Driessen, 2019). The Pa5 had strong labeling of afferents in Pirt-tdT, 5-HT3-tdT, TRPV1-tdT, and Tac1-tdT, although all four markers also labeled subsets of trigeminal afferent terminations within the Sp5. As such the genetic reporters cannot link Pa5 labeling with vagal afferents specifically. Intraganglionic injection of AAV9-flex-GFP labeled vagal TRPV1^+^ and Tac1^+^ terminations within the ipsilateral Pa5, confirming that vagal afferent subsets directly innervate this non-nTS area within the medulla (although it should be noted that the peripheral organs innervated by these subsets have not been identified in this study). The observation in our AAV9 studies of large numbers of ROSA 26-mediated tdTomato^+^ fibers lacking AAV9-mediated GFP in the Pa5 suggest the additional presence of nonvagal TRPV1^+^ and Tac1^+^ fibers, which likely originate from trigeminal afferents. It is likely that the vagal Pa5 terminations include Tac1^+^/TRPV1^+^ jugular afferents, but we presently cannot determine whether nodose TRPV1^+^ afferents also innervate the Pa5. In addition, we found a subset of TRPV1^−^ vagal afferents innervated the Pa5, but it is also unclear whether these are a subset of the vagal Tac1^+^ terminations. It should be emphasized that we observed the majority of Tac1^+^ vagal afferents (many of which are jugular TRPV1^+^ neurons) terminating in the nTS. This contradicts recent retrograde tracing data from the nTS and Pa5 in the rat, which suggested that jugular fibers only terminate in the Pa5 (Driessen et al., 2015; McGovern et al., 2015b). Importantly, however, we failed to detect any terminations in the Pa5 from lung-specific TRPV1^+^ or Tac1^+^ afferents (identified using rAAV2 instilled into the airways). This is in disagreement with McGovern et al. (2015a,b), who showed that herpes simplex virus vectors injected into the trachea or the lung in the rat labeled the Pa5, although the labeling from the lung was limited. The rAAV2 used in this study was instilled into the airways via intubation, and this may have preferentially labeled lower airways afferents rather than tracheal afferents. Further anatomic and physiological study is clearly needed to clarify the role of vagal, trigeminal, and potentially other afferent terminations within the Pa5 in mediating respiratory reflexes.

Last, we noted significant vagal afferent bilateral innervation of both the area postrema and the DMX, consistent with previous tracing experiments from the nodose ganglia (Kalia and Sullivan, 1982; Neuhuber and Sandoz, 1986). No labeling of the nucleus ambiguus or the hypoglossal nucleus was noted. The area postrema, which controls emesis, was most strongly labeled by TRPV1^+^ afferents, with only sparse labeling by either TRPV1^−^ or Tac1^+^ vagal afferents. This suggests that the majority of this innervation is via nodose C-fibers. Vagal afferents play an important role in emesis (Babic and Browning, 2014), and electrophysiological data suggest that vagal C-fibers terminate within the area postrema (Kubin et al., 1991). Vagal afferent terminations in the DMX, the premotor nucleus for parasympathetic innervation of the gut, were rare, but we found evidence of TRPV1^+^, Tac1^+^, and TRPV1^−^ fibers. No lung-specific fibers were identified innervating the DMX. Previous studies have identified direct innervation of DMX by abdominal vagal afferents, but there is little known about their role distinct from the well established vagal afferent–nTS–DMX networks involved in autonomic regulation (Rinaman et al., 1989; Renehan et al., 1995; Mussa and Verberne, 2013).

The reporters expressed in these studies (tdTomato and GFP) were soluble proteins that diffused along the entire length of the afferents (including soma, axons, and terminals). Thus, it is not possible to definitively determine the difference between axonal and terminal structures within serial sections of the medulla. As such, the presence of a reporter-expressing fiber within a particular area does not definitively indicate functional connectivity within that region. Nevertheless, high-magnification *z*-stack images show that boutons (sometimes in close proximity to other neuronal soma) are found along much of the reporter-expressing fibers as they invade the nTS, Pa5, area postrema, and DMX. It is possible that each afferent fiber innervates a large number of second-order neurons, but this cannot be rigorously assessed without using a reporter targeted to synapses.

In summary, this study used cell-specific reporter expression to identify the brainstem pathways of functionally distinct vagal afferent subsets in the mouse. Our data indicate that TRPV1^+^ vagal afferents innervate the ipsilateral and contralateral dorsal/medial nTS subnuclei and the ipsilateral paratrigeminal complex, whereas TRPV1^−^ vagal afferents innervate the ipsilateral rostral/ventral/lateral nTS subnuclei in addition to the ipsilateral paratrigeminal complex. The differences in central terminations by specific afferent subsets likely provide a neuroanatomical substrate for subset-specific reflexes.

## Synthesis

Reviewing Editor: Darcy Kelley, Columbia University Decisions are customarily a result of the Reviewing Editor and the peer reviewers coming together and discussing their recommendations until a consensus is reached. When revisions are invited, a fact-based synthesis statement explaining their decision and outlining what is needed to prepare a revision will be listed below. The following reviewer(s) agreed to reveal their identity: Lu-Yuan Lee.

Overall assessment:

The authors utilise cre-expressing (Pirt-Cre, TRPV1-Cre, 5HT3-Cre and Tac1-Cre) mice where these gene products are known to be expressed in peripheral sensory neurons. These animals were then crossed with a floxed tdTomato line to visualise these neurons in the nodose/jugular ganglia and their central axons in the brainstem. These expression patterns were compared. In addition the authors utilised immunohistochemistry for TRPV1, TrkA and B to assess the degree of co-expression across cohorts. As the authors illustrate, but do not explicitly state is all cases, these promoters are utilised in other sensory neurons too, trigeminal, geniculate, pertrosal all of which terminate at regions overlapping nodose/jugular neurons either within the NTS or Pa5. In addition, the presence of intrinsic CNS neurons that at one time utilised the promoter also expressed tdTomato. These experiments largely confirmed previous findings with respect to expression patterns within the nodose/jugular ganglia. The central axons and their termination are not able to be isolated in these tdTomato animals. This first aspect of the manuscript provides a good resource for the cre animals where few reports of these brainstem expression patterns exist. The authors recognised a major disadvantage in the cre-lines in that fluorescent protein persists even after the promoter is not longer utilised in the given neuron (lines 387-390). This could perhaps be stated closer to the beginning of the results. The second aspect of the study gains greater specificity in that a flex virus in injected into the nodose ganglion of the TRPV1-Cre and Tac1-Cre lines or into the internal airways. Here the nodose/jugular axon and terminal fields are more precisely mapped. This is where most of the novel data is presented and the results are very interesting though many details about the success or not of these efforts are not reported.

While, the results of this study reported the mapping of central projection of vagal afferents innervating all visceral organs, but a special focus appeared to be placed on “lung-specific” vagal afferents (pages 8, 25, 26; Fig 20), . Authors may want to distinguish more clearly between them in the presentation and interpretation of data.

For example, the finding that the lung-specific TRPV1+ and Tac1+ afferents’ central projections are restricted to the caudal medial nTS, with no innervation of any other medulla regions, is particularly interesting because the vagal TRPV1+ and Tac1+ afferents labeled with AAV9-flex-GFP terminate within the ipsilateral paratrigeminal complex (Pa5) (page 28).

It should be interesting to know if these observations on lung-specific vagal afferents bear any important implications in the elicitation of certain respiratory reflex responses such as cough, tachypnea, etc.

It is understood that the Cre-expressing animal models are only available in the mouse. Authors may want to discuss if the findings in this study can be expanded to other species that were more extensively used in the study of regulatory functions of vagal afferents (e.g., rat, cat).

In this study, the lung-specific vagal afferent terminations were identified by the intratracheal instillation of rAAV (page 8). Has it been verified that the virus/MEM instilled stayed within the respiratory tract (e.g., not being coughed out and swallowed into gastroesophageal tract)?

Major points:

Classifying sensory neurons as nociceptors because they express TRPV1 is not helpful. Particular when the spinal visceral afferents convey such signals. Using this term, nociceptor, implies vagal sensory neurons mediate a signal that will be perceived as pain. One could argue the input to Pa5 may mediate a pain signal, but this has not been demonstrated. Yet most/all other homeostatic signals that reach the NTS are not pain related. Eg: C-fibre baroreceptors convey high blood pressure and the signals are not perceived. The category terms nociceptor and non-nociceptor should be abolished from the article, TRPV1+ or TRPV1- should be used instead as is specific to what was detected/expressed.

Across experiments it is not clear how many animals were used in each experiment. The data is neuroanatomical in nature but at the very least list the numbers used should be detailed in the methods. It is not clear how many times the airway administration was tried, one thing of interest here is the success rate of viral delivery in this way. Peripheral infection with viral tools has proved very difficult across labs. These practical details are missing yet are of interest. Questions abound regarding the peripheral administration of viral vectors. Was there any difference in outcomes between upper and lower airway attempts. How many nodose neurons were infected across animals? The tracing shown in Figure 20 is a result of how many nodose neurons? Greater detail about the success or not of these experiments should be reported in the results around Figure 20.

The florescent makers are labelling all aspects of the sensory (and other intrinsic) neurons and while this produces the best signal to noise one disadvantage is the axons and terminals cannot be differentiated. Thus where the sensory neurons are terminating may be different to where they travel, ie axons of passage versus synaptic terminals. This limitation needs to be addressed, case in point with regard to the DMV, are these sensory terminals or axons of passage at the DMV?

Minor points:

-What is the significance of the different size cell bodies; Fig 3. Explain exactly how cell diameter was determined as the range of 7-30 um does not seem true from Figure 1 inserts? Where only full completely imaged neurons measured or in 2D only?

-Line 260, unabbreviated IF in all instances. Line 390, Line 393, unabbreviated IR.

-Line 267, ratios need two numbers, I assume here x:1 in all cases?

-Line 354-356, refer to Fig 1 and 2 as reflects differential ganglia expression.

-Line 417, give rational for the co-injection.

-Line 434, referring to length of axons?

-Lines 436, 437, 439, 452; use of ‘nerves’ confusing, intrinsic nerves or nodose/sensory axons/terminals?

-Line 472, how many nodose neurons?

-Line 476, distances relative to obex?

-Line 502-4, the authors cannot claim this as no reflexes were tested/initiated.

Figures:

F8, 9, 10, and 11, label with the cre line.

F17, 18 19 and 20, identify the colour scheme as in previous Figure panels.

Minor points:

-What is the significance of the different size cell bodies; Fig 3. Explain exactly how cell diameter was determined as the range of 7-30 um does not seem true from Figure 1 inserts? Where only full completely imaged neurons measured or in 2D only?

-Line 260, unabbreviated IF in all instances. Line 390, Line 393, unabbreviated IR.

-Line 267, ratios need two numbers, I assume here x:1 in all cases?

-Line 354-356, refer to Fig 1 and 2 as reflects differential ganglia expression.

-Line 417, give rational for the co-injection.

-Line 434, referring to length of axons?

-Lines 436, 437, 439, 452; use of ‘nerves’ confusing, intrinsic nerves or nodose/sensory axons/terminals?

-Line 472, how many nodose neurons?

-Line 473: “Fig 20A and not shown”. What does it mean?

-Line 476, distances relative to obex?

-Line 502-4, the authors cannot claim this as no reflexes were tested/initiated.

Figures:

F8, 9, 10, and 11, label with the cre line.

F17, 18 19 and 20, identify the colour scheme as in previous Figure panels.

## References

[B1] Babic T, Browning KN (2014) The role of vagal neurocircuits in the regulation of nausea and vomiting. Eur J Pharmacol 722:38–47. 10.1016/j.ejphar.2013.08.047 24184670 PMC3893663

[B2] Bonham AC, McCrimmon DR (1990) Neurones in a discrete region of the nucleus tractus solitarius are required for the Breuer-Hering reflex in rat. J Physiol 427:261–280. 10.1113/jphysiol.1990.sp018171 2213599 PMC1189930

[B3] Carr MJ, Undem BJ (2003) Bronchopulmonary afferent nerves. Respirology 8:291–301. 10.1046/j.1440-1843.2003.00473.x 14528878

[B4] Carter MS, Krause JE (1990) Structure, expression, and some regulatory mechanisms of the rat preprotachykinin gene encoding substance P, neurokinin A, neuropeptide K, and neuropeptide gamma. J Neurosci 10:2203–2214. 1695945 10.1523/JNEUROSCI.10-07-02203.1990 PMC6570392

[B5] Caterina MJ, Schumacher MA, Tominaga M, Rosen TA, Levine JD, Julius D (1997) The capsaicin receptor: a heat-activated ion channel in the pain pathway. Nature 389:816–824. 10.1038/39807 9349813

[B6] Cavanaugh DJ, Chesler AT, Jackson AC, Sigal YM, Yamanaka H, Grant R, O'Donnell D, Nicoll RA, Shah NM, Julius D, Basbaum AI (2011) Trpv1 reporter mice reveal highly restricted brain distribution and functional expression in arteriolar smooth muscle cells. J Neurosci 31:5067–5077. 10.1523/JNEUROSCI.6451-10.2011 21451044 PMC3087977

[B7] Chang RB, Strochlic DE, Williams EK, Umans BD, Liberles SD (2015) Vagal Sensory Neuron Subtypes that Differentially Control Breathing. Cell 161:622–633. 10.1016/j.cell.2015.03.022 25892222 PMC4842319

[B8] Chou YL, Scarupa MD, Mori N, Canning BJ (2008) Differential effects of airway afferent nerve subtypes on cough and respiration in anesthetized guinea pigs. Am J Physiol Regul Integr Comp Physiol 295:R1572–1584. 10.1152/ajpregu.90382.2008 18768768 PMC4060899

[B9] Chou YL, Mori N, Canning BJ (2018) Opposing effects of bronchopulmonary C-fiber subtypes on cough in guinea pigs. Am J Physiol Regul Integr Comp Physiol 314:R489–R498. 10.1152/ajpregu.00313.2017 29187382 PMC5899253

[B10] Chuaychoo B, Lee MG, Kollarik M, Undem BJ (2005) Effect of 5-hydroxytryptamine on vagal C-fiber subtypes in guinea pig lungs. Pulm Pharmacol Ther 18:269–276. 10.1016/j.pupt.2004.12.010 15777609

[B11] Davies RO, Kubin L (1986) Projection of pulmonary rapidly adapting receptors to the medulla of the cat: an antidromic mapping study. J Physiol 373:63–86. 10.1113/jphysiol.1986.sp016035 3746682 PMC1182525

[B12] Davies RO, Kubin L, Pack AI (1987) Pulmonary stretch receptor relay neurones of the cat: location and contralateral medullary projections. J Physiol 383:571–585. 10.1113/jphysiol.1987.sp016429 3656136 PMC1183090

[B13] Donoghue S, Garcia M, Jordan D, Spyer KM (1982) The brain-stem projections of pulmonary stretch afferent neurones in cats and rabbits. J Physiol 322:353–363. 10.1113/jphysiol.1982.sp014041 7069621 PMC1249674

[B14] Driessen AK (2019) Vagal afferent processing by the paratrigeminal nucleus. Front Physiol 10:1110. 10.3389/fphys.2019.01110 31555145 PMC6722180

[B15] Driessen AK, Farrell MJ, Mazzone SB, McGovern AE (2015) The role of the paratrigeminal nucleus in vagal afferent evoked respiratory reflexes: a neuroanatomical and functional study in guinea pigs. Front Physiol 6:378. 10.3389/fphys.2015.00378 26733874 PMC4685097

[B16] Ho CY, Gu Q, Lin YS, Lee LY (2001) Sensitivity of vagal afferent endings to chemical irritants in the rat lung. Respir Physiol 127:113–124. 10.1016/s0034-5687(01)00241-9 11504584

[B17] Hooper JS, Hadley SH, Morris KF, Breslin JW, Dean JB, Taylor-Clark TE (2016) Characterization of cardiovascular reflexes evoked by airway stimulation with allylisothiocyanate, capsaicin, and ATP in Sprague-Dawley rats. J Appl Physiol 120:580–591. 10.1152/japplphysiol.00944.2015 26718787 PMC4868373

[B18] Hooper JS, Stanford KR, Alencar PA, Alves NG, Breslin JW, Dean JB, Morris KF, Taylor-Clark TE (2019) Nociceptive pulmonary–cardiac reflexes are altered in the spontaneously hypertensive rat. J Physiol 597:3255–3279. 10.1113/JP278085 31077371 PMC6602842

[B19] Kalia M, Mesulam MM (1980) Brain stem projections of sensory and motor components of the vagus complex in the cat: I. The cervical vagus and nodose ganglion. J Comp Neurol 193:435–465. 10.1002/cne.901930210 7440777

[B20] Kalia M, Sullivan JM (1982) Brainstem projections of sensory and motor components of the vagus nerve in the rat. J Comp Neurol 211:248–265. 10.1002/cne.902110304 7174893

[B21] Kim YS, Anderson M, Park K, Zheng Q, Agarwal A, Gong C, Saijilafu, Young L, He S, LaVinka PC, Zhou F, Bergles D, Hanani M, Guan Y, Spray DC, Dong X (2016) Coupled activation of primary sensory neurons contributes to chronic pain. Neuron 91:1085–1096. 10.1016/j.neuron.2016.07.044 27568517 PMC5017920

[B22] Kubin L, Kimura H, Davies RO (1991) The medullary projections of afferent bronchopulmonary C fibres in the cat as shown by antidromic mapping. J Physiol 435:207–228. 10.1113/jphysiol.1991.sp018506 1770435 PMC1181458

[B23] Kubin L, Alheid GF, Zuperku EJ, McCrimmon DR (2006) Central pathways of pulmonary and lower airway vagal afferents. J Appl Physiol 101:618–627. 10.1152/japplphysiol.00252.2006 16645192 PMC4503231

[B24] Kupari J, Haring M, Agirre E, Castelo-Branco G, Ernfors P (2019) An atlas of vagal sensory neurons and their molecular specialization. Cell Rep 27:2508–2523.e4. 10.1016/j.celrep.2019.04.096 31116992 PMC6533201

[B25] Lawson SN, Perry MJ, Prabhakar E, McCarthy PW (1993) Primary sensory neurones: neurofilament, neuropeptides, and conduction velocity. Brain Res Bull 30:239–243. 10.1016/0361-9230(93)90250-f 7681350

[B26] Lieu T, Kollarik M, Myers AC, Undem BJ (2011) Neurotrophin and GDNF family ligand receptor expression in vagal sensory nerve subtypes innervating the adult guinea pig respiratory tract. Am J Physiol Lung Cell Mol Physiol 300:L790–L798. 10.1152/ajplung.00449.2010 21335521 PMC3284280

[B27] Lipski J, Ezure K, Wong She RB (1991) Identification of neurons receiving input from pulmonary rapidly adapting receptors in the cat. J Physiol 443:55–77. 10.1113/jphysiol.1991.sp018822 1822538 PMC1179830

[B28] Mazzone SB, Geraghty DP (1999) Respiratory action of capsaicin microinjected into the nucleus of the solitary tract: involvement of vanilloid and tachykinin receptors. Br J Pharmacol 127:473–481. 10.1038/sj.bjp.0702522 10385248 PMC1566015

[B29] Mazzone SB, Geraghty DP (2000) Respiratory actions of tachykinins in the nucleus of the solitary tract: characterization of receptors using selective agonists and antagonists. Br J Pharmacol 129:1121–1131. 10.1038/sj.bjp.0703172 10725260 PMC1571949

[B30] Mazzone SB, Canning BJ (2002) Synergistic interactions between airway afferent nerve subtypes mediating reflex bronchospasm in guinea pigs. Am J Physiol Regul Integr Comp Physiol 283:R86–R98. 10.1152/ajpregu.00007.2002 12069934

[B31] Mazzone SB, Mori N, Canning BJ (2005) Synergistic interactions between airway afferent nerve subtypes regulating the cough reflex in guinea-pigs. J Physiol 569:559–573. 10.1113/jphysiol.2005.093153 16051625 PMC1464254

[B32] Mazzone SB, Tian L, Moe AAK, Trewella MW, Ritchie ME, McGovern AE (2020) Transcriptional profiling of individual airway projecting vagal sensory neurons. Mol Neurobiol 57:949–963. 31630330 10.1007/s12035-019-01782-8

[B33] McGovern AE, Davis-Poynter N, Yang SK, Simmons DG, Farrell MJ, Mazzone SB (2015a) Evidence for multiple sensory circuits in the brain arising from the respiratory system: an anterograde viral tract tracing study in rodents. Brain Struct Funct 220:3683–3699. 10.1007/s00429-014-0883-9 25158901

[B34] McGovern AE, Driessen AK, Simmons DG, Powell J, Davis-Poynter N, Farrell MJ, Mazzone SB (2015b) Distinct brainstem and forebrain circuits receiving tracheal sensory neuron inputs revealed using a novel conditional anterograde transsynaptic viral tracing system. J Neurosci 35:7041–7055. 10.1523/JNEUROSCI.5128-14.2015 25948256 PMC6605260

[B35] Mussa BM, Verberne AJ (2013) The dorsal motor nucleus of the vagus and regulation of pancreatic secretory function. Exp Physiol 98:25–37. 10.1113/expphysiol.2012.066472 22660814

[B36] Nassenstein C, Taylor-Clark TE, Myers AC, Ru F, Nandigama R, Bettner W, Undem BJ (2010) Phenotypic distinctions between neural crest and placodal derived vagal C-fibres in mouse lungs. J Physiol 588:4769–4783. 10.1113/jphysiol.2010.195339 20937710 PMC3010145

[B37] Neuhuber WL, Sandoz PA (1986) Vagal primary afferent terminals in the dorsal motor nucleus of the rat: are they making monosynaptic contacts on preganglionic efferent neurons? Neurosci Lett 69:126–130. 10.1016/0304-3940(86)90590-2 2429236

[B38] Patapoutian A, Tate S, Woolf CJ (2009) Transient receptor potential channels: targeting pain at the source. Nat Rev Drug Discov 8:55–68. 10.1038/nrd2757 19116627 PMC2755576

[B39] Patel KN, Liu Q, Meeker S, Undem BJ, Dong X (2011) Pirt, a TRPV1 modulator, is required for histamine-dependent and -independent itch. PLoS One 6:e20559. 10.1371/journal.pone.0020559 21655234 PMC3105090

[B40] Patil MJ, Hovhannisyan AH, Akopian AN (2018) Characteristics of sensory neuronal groups in CGRP-cre-ER reporter mice: comparison to Nav1.8-cre, TRPV1-cre and TRPV1-GFP mouse lines. PLoS One 13:e0198601. 10.1371/journal.pone.0198601 29864146 PMC5986144

[B41] Paxinos G, Franklin B (2012) Mouse brain in stereotaxic coordinates, Ed 4. Amsterdam: Elsevier.

[B42] Potenzieri C, Meeker S, Undem BJ (2012) Activation of mouse bronchopulmonary C-fibers by serotonin and allergen-ovalbumin challenge. J Physiol 590:5449–5459. 22907059 10.1113/jphysiol.2012.237115 PMC3515830

[B43] Renehan WE, Zhang X, Beierwaltes WH, Fogel R (1995) Neurons in the dorsal motor nucleus of the vagus may integrate vagal and spinal information from the GI tract. Am J Physiol 268:G780–G790. 10.1152/ajpgi.1995.268.5.G780 7762662

[B44] Ricco MM, Kummer W, Biglari B, Myers AC, Undem BJ (1996) Interganglionic segregation of distinct vagal afferent fibre phenotypes in guinea-pig airways. J Physiol 496:521–530. 10.1113/jphysiol.1996.sp021703 8910234 PMC1160895

[B45] Rinaman L, Card JP, Schwaber JS, Miselis RR (1989) Ultrastructural demonstration of a gastric monosynaptic vagal circuit in the nucleus of the solitary tract in rat. J Neurosci 9:1985–1996. 10.1523/JNEUROSCI.09-06-01985.1989 2723763 PMC6569733

[B46] Robinson DR, Gebhart GF (2008) Inside information: the unique features of visceral sensation. Mol Interv 8:242–253. 10.1124/mi.8.5.9 19015388 PMC2732716

[B47] Sherrington C (1906) The integrative action of the nervous system. New Haven: Scribner.

[B48] Stanford KR, Hadley SH, Barannikov I, Ajmo JM, Bahia PK, Taylor-Clark TE (2019) Antimycin A-induced mitochondrial dysfunction activates vagal sensory neurons via ROS-dependent activation of TRPA1 and ROS-independent activation of TRPV1. Brain Res 1715:94–105. 10.1016/j.brainres.2019.03.029 30914247 PMC6500470

[B49] Surdenikova L, Ru F, Nassenstein C, Tatar M, Kollarik M (2012) The neural crest- and placodes-derived afferent innervation of the mouse esophagus. Neurogastroenterol Motil 24:e517–e525. 10.1111/nmo.12002 22937918

[B50] Thoren P (1979) Role of cardiac vagal C-fibers in cardiovascular control. Rev Physiol Biochem Pharmacol 86:1–94. 386467 10.1007/BFb0031531

[B51] Treweek JB, Chan KY, Flytzanis NC, Yang B, Deverman BE, Greenbaum A, Lignell A, Xiao C, Cai L, Ladinsky MS, Bjorkman PJ, Fowlkes CC, Gradinaru V (2015) Whole-body tissue stabilization and selective extractions via tissue-hydrogel hybrids for high-resolution intact circuit mapping and phenotyping. Nat Protoc 10:1860–1896. 10.1038/nprot.2015.122 26492141 PMC4917295

[B52] Undem BJ, Chuaychoo B, Lee MG, Weinreich D, Myers AC, Kollarik M (2004) Subtypes of vagal afferent C-fibres in guinea-pig lungs. J Physiol 556:905–917. 10.1113/jphysiol.2003.060079 14978204 PMC1665007

[B53] Usoskin D, Furlan A, Islam S, Abdo H, Lönnerberg P, Lou D, Hjerling-Leffler J, Haeggström J, Kharchenko O, Kharchenko PV, Linnarsson S, Ernfors P (2015) Unbiased classification of sensory neuron types by large-scale single-cell RNA sequencing. Nat Neurosci 18:145–153. 10.1038/nn.3881 25420068

[B54] Wang J, Kollarik M, Ru F, Sun H, McNeil B, Dong X, Stephens G, Korolevich S, Brohawn P, Kolbeck R, Undem B (2017) Distinct and common expression of receptors for inflammatory mediators in vagal nodose versus jugular capsaicin-sensitive/TRPV1-positive neurons detected by low input RNA sequencing. PLoS One 12:e0185985. 10.1371/journal.pone.0185985 28982197 PMC5628920

[B55] Yu Y, Bradley A (2001) Engineering chromosomal rearrangements in mice. Nat Rev Genet 2:780–790. 10.1038/35093564 11584294

[B56] Yu S, Undem BJ, Kollarik M (2005) Vagal afferent nerves with nociceptive properties in guinea-pig oesophagus. J Physiol 563:831–842. 10.1113/jphysiol.2004.079574 15649987 PMC1665603

[B57] Zhuo H, Ichikawa H, Helke CJ (1997) Neurochemistry of the nodose ganglion. Prog Neurobiol 52:79–107. 10.1016/s0301-0082(97)00003-8 9185234

